# CRISPR-RNAa: targeted activation of translation using dCas13 fusions to translation initiation factors

**DOI:** 10.1093/nar/gkac680

**Published:** 2022-08-11

**Authors:** Peter B Otoupal, Brady F Cress, Jennifer A Doudna, Joseph S Schoeniger

**Affiliations:** Biomanufacturing and Biomaterials Department, Sandia National Laboratories, Livermore, CA, USA; Deconstruction Division, Joint BioEnergy Institute, Lawrence Berkeley National Laboratory, Emeryville, CA, USA; Agile BioFoundry, Department of Energy, Emeryville, CA, USA; Innovative Genomics Institute, University of California, Berkeley, CA, USA; Department of Molecular and Cell Biology, University of California, Berkeley, CA, USA; Innovative Genomics Institute, University of California, Berkeley, CA, USA; Department of Molecular and Cell Biology, University of California, Berkeley, CA, USA; California Institute for Quantitative Biosciences, University of California, Berkeley, CA, USA; Department of Chemistry, University of California, Berkeley, CA, USA; Howard Hughes Medical Institute, University of California, Berkeley, CA, USA; Molecular Biophysics & Integrated Bioimaging Division, Lawrence Berkeley National Laboratory, Berkeley, CA, USA; Gladstone Institutes, University of California, San Francisco, CA, USA; Systems Biology Department, Sandia National Laboratories, Livermore, CA, USA

## Abstract

Tools for synthetically controlling gene expression are a cornerstone of genetic engineering. CRISPRi and CRISPRa technologies have been applied extensively for programmable modulation of gene transcription, but there are few such tools for targeted modulation of protein translation rates. Here, we employ CRISPR-Cas13 as a programmable activator of translation. We develop a novel variant of the catalytically-deactivated Cas13d enzyme dCasRx by fusing it to translation initiation factor IF3. We demonstrate dCasRx-IF3’s ability to enhance expression 21.3-fold above dCasRx when both are targeted to the start of the 5′ untranslated region of mRNA encoding red fluorescent protein in *Escherichia coli*. Activation of translation is location-dependent, and we show dCasRx-IF3 represses translation when targeted to the ribosomal binding site, rather than enhancing it. We provide evidence that dCasRx-IF3 targeting enhances mRNA stability relative to dCasRx, providing mechanistic insights into how this new tool functions to enhance gene expression. We also demonstrate targeted upregulation of native LacZ 2.6-fold, showing dCasRx-IF3’s ability to enhance expression of endogenous genes. dCasRx-IF3 requires no additional host modification to influence gene expression. This work outlines a novel approach, CRISPR-RNAa, for post-transcriptional control of translation to activate gene expression.

## INTRODUCTION

Molecular tools derived from the prokaryotic adaptive immune system, CRISPR, have revolutionized the biological sciences. Exploitation of CRISPR-associated (Cas) nucleases alongside short, easily modifiable CRISPR RNA (crRNA) guides (1), enables researchers to target Cas proteins rapidly and reliably to specific genetic loci. By introducing mutations to catalytically inactivate these nucleases, and tethering additional functional domains, researchers have repurposed dCas proteins into a suite of tools for the precise manipulation of gene expression.

CRISPR interference (CRISPRi) allows for the targeted downregulation of gene expression through targeted DNA-binding and transcriptional inhibition, and is well established in both prokaryotic (2,3) and eukaryotic (4) organisms. Analogously, CRISPR activation (CRISPRa) tools enable the selective enhancement of transcription rates of target genes (5,6). In eukaryotes, fusing transcriptional regulators such as VP64 or VPR to dCas9 or dCpf1 allows for targeted gene expression enhancement via recruitment of RNA polymerase (7–9). In prokaryotes, researchers have similarly employed fusions of dCas9 to the omega subunit of RNA polymerase to enhance transcription (10). CRISPRa in prokaryotic systems has typically resulted in weaker effects on the level of ∼10-fold, and may require concurrent downregulation of native genes such as *rpoZ* to achieve substantial effects (11–18). While more recent work has improved the fold of gene-expression enhancement obtainable with CRISPRa in bacteria (11,12,19–24), efficacy has typically lagged behind the robust enhancement of gene expression in eukaryotic systems.

Recently, researchers uncovered a family of Type VI CRISPR nucleases (dubbed Cas13) that target RNA rather than DNA (25,26), facilitating the development of synthetic biology technologies for targeted manipulation of transcripts beyond targeted cleavage. By inactivating their nuclease activities and tethering them to functional domains, dCas13 (‘dead’ Cas13) proteins have been repurposed for a wide variety of applications, including editing RNA sequences (27–29), imaging RNA localization (30), exploring alternative splicing (31), and combating the SARS-CoV-2 pandemic (32,33). Researchers have also employed dCas13 for targeted repression of gene expression through inhibition of translation (34), in a fashion similar to CRISPRi transcriptional inhibition.

Because technologies for facile and programmable targeted activation of translation are lacking, we sought to convert dCas13 into a tool for programmable activation of translation to enhance protein expression. We hypothesized that tethering dCas13 to a functional domain involved in translation would create a tool for selectively increasing the translation rate of a target mRNA. Specifically, because the rate limiting step of translation is initiation in both prokaryotes (35,36) and eukaryotes (37–39), we reasoned that tethering translation initiation factors to dCas13 might facilitate enhanced translation initiation at a targeted ribosome binding site. In *Escherichia coli*, three initiation factors (IF1, IF2 and IF3, encoded by the genes *infA*, *infB* and *infC* respectively) are involved in commencing translation. IF1 and IF3 work together to perform the first step of translation initiation, in which they bind to the 30S subunit and prevent 70S ribosome assembly until the remaining machinery is in place (40,41). IF1 and IF3 are also substantially smaller (72 and 180 amino acids respectively) than IF2 (890 amino acids). We therefore hypothesized that either IF1 or IF3 fusions with dCas13 would be promising candidates for a tool to enhance translation.

We show here that both IF1 and IF3 fusions to dCas13 significantly activate expression of red fluorescent protein (RFP) in comparison to dCas13 alone, with the IF3 fusion providing elevated activation relative to IF1. We term this effect CRISPR-RNA activation (CRISPR-RNAa). Enhancement of gene expression is observed in the context of strains with unmodified translation machinery, exhibiting only a small increase in lag time with no other fitness impacts. We explore a library of 36 crRNAs to show that dCas13-IF3 activation is position-dependent, and is greater than the effects of crRNAs or dCas13 alone. We demonstrate a dose-dependent response of the tool, with greater IF-specific effects at higher target mRNA expression levels. Furthermore, we show that reducing temperature enhances the IF-specific effects further, allowing us to obtain levels of gene expression activation comparable to those obtained with analogous tools for transcriptional activation. We provide evidence that this fluorescence increase is correlated with an increase in mRNA stability, presumably by increasing translation initiation. Finally, we apply CRISPR-RNAa to activate expression of native β-galactosidase, highlighting its broader applicability. These results demonstrate how CRISPR-RNAa is a simple and modular tool for programmable activation of gene translation that, while still nascent, opens a new avenue for programmable enhancement of gene expression.

## MATERIALS AND METHODS

### Strains

*Escherichia coli* strains were used for all experiments and plasmid construction. For plasmid construction, transformations were performed using strain NEB^®^ 5-alpha Competent *E. coli* (New England BioLabs). For β-galactosidase experiments, a derivative of strain DH1 with an intact native Lac operon, AG1 competent cells (Agilent), was used. For fluorescence experiments, a DH1 strain containing approximately sixty-six copies of *rfp* encoding Red Fluorescent Protein (RFP) under control of the IPTG-inducible *lacUV5* integrated at the *intA* loci was used (42). This strain was recovered from the private instance of the Joint BioEnergy Institute Registry under the JBEI ID JBx_078907, and is also available in the public instance of the Joint BioEnergy Institute Registry under the ID JPUB_010181 (https://public-registry.jbei.org/folders/354). Plasmids encoding CRISPR machinery were transformed into these base strains to create the experimental strains used in this study. A list of all strains can be found in Supplementary Table S1.

### Culture conditions

**Figure 1.**
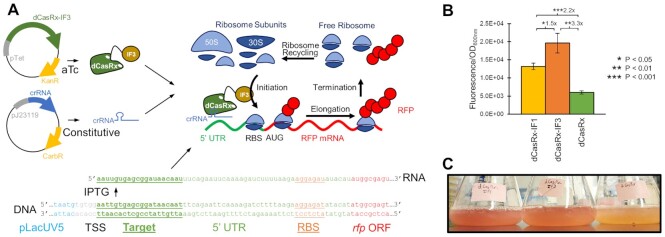
Targeted activation of mRNA translation with dCasRx-IF fusions. (**A**) A two-plasmid system encoding aTc-inducible dCasRx or dCasRx-IF fusions on the first plasmid and a constitutively expressed crRNA on the second plasmid is expressed in *E. coli* harboring IPTG-inducible *rfp*. The crRNAs guide the dCasRx variants to the start of the 5′ UTR, which subsequently recruits ribosomal subunits to the target mRNA to presumably increase translation initiation. The target 20nt crRNA location is underlined. (**B**) RFP fluorescence of *E. coli* expressing *rfp*-targeting crRNA alongside dCasRx variants. Error bars represent the standard deviation of biological triplicates. Asterisks indicate *P*-values of two-tailed type II Student's *t*-tests. (**C**) Cultures of strains with *rfp*-targeting dCasRx-IF1 (left) and dCasRx-IF3 (middle) relative to dCasRx (right).

**Figure 2.**
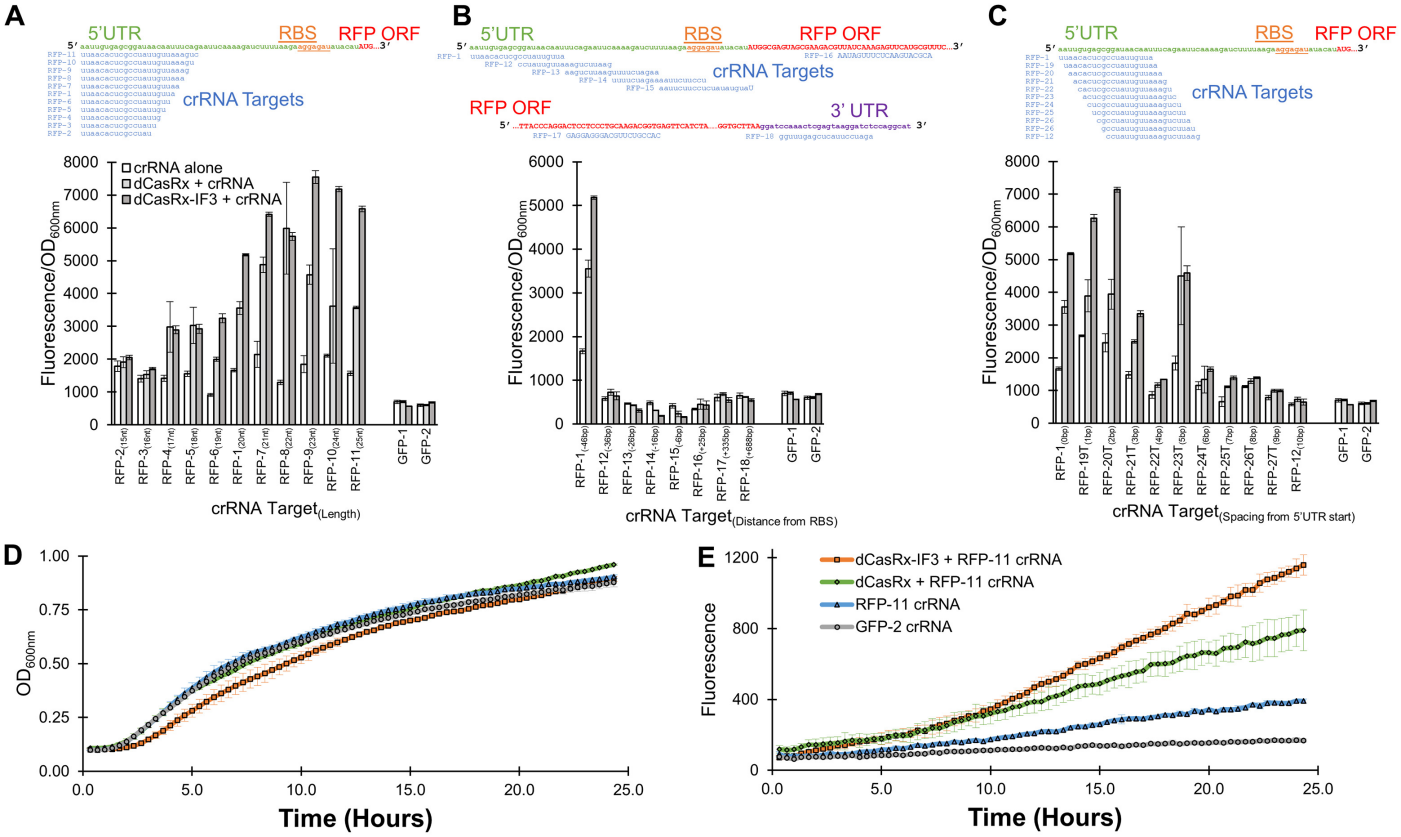
IF3 fusion to dCasRx drives maximal translation. (**A**) Effect of varying the length of the 20nt crRNA target ‘RFP-1’ used in Figure 1, from 15 to 25 nts. mRNA structure and crRNA target location are shown above, and normalized fluorescence measurements below. crRNA length is shown in subscripts next to each crRNA name. (**B**) Effect of varying the target location of the crRNA across the mRNA. Distance between the start of the ‘UTR. (**C**) Effect of shifting the crRNA target location 1 nt at a time away from the start of the 5′ UTR. Distance between the RBS and the 5′ *rfp* mRNA target location are shown in subscripts next to each crRNA name. In A–C, two variants of GFP-targeting crRNAs are included as controls. Target distances from the start of the *rfp* mRNA 5′ start are shown in subscripts next to each crRNA name. Kinetic (**D**) growth and **(E**) fluorescence measurements of strains expressing the noted CRISPR machinery. All error bars represent the standard deviation of biological triplicates.

**Figure 3.**
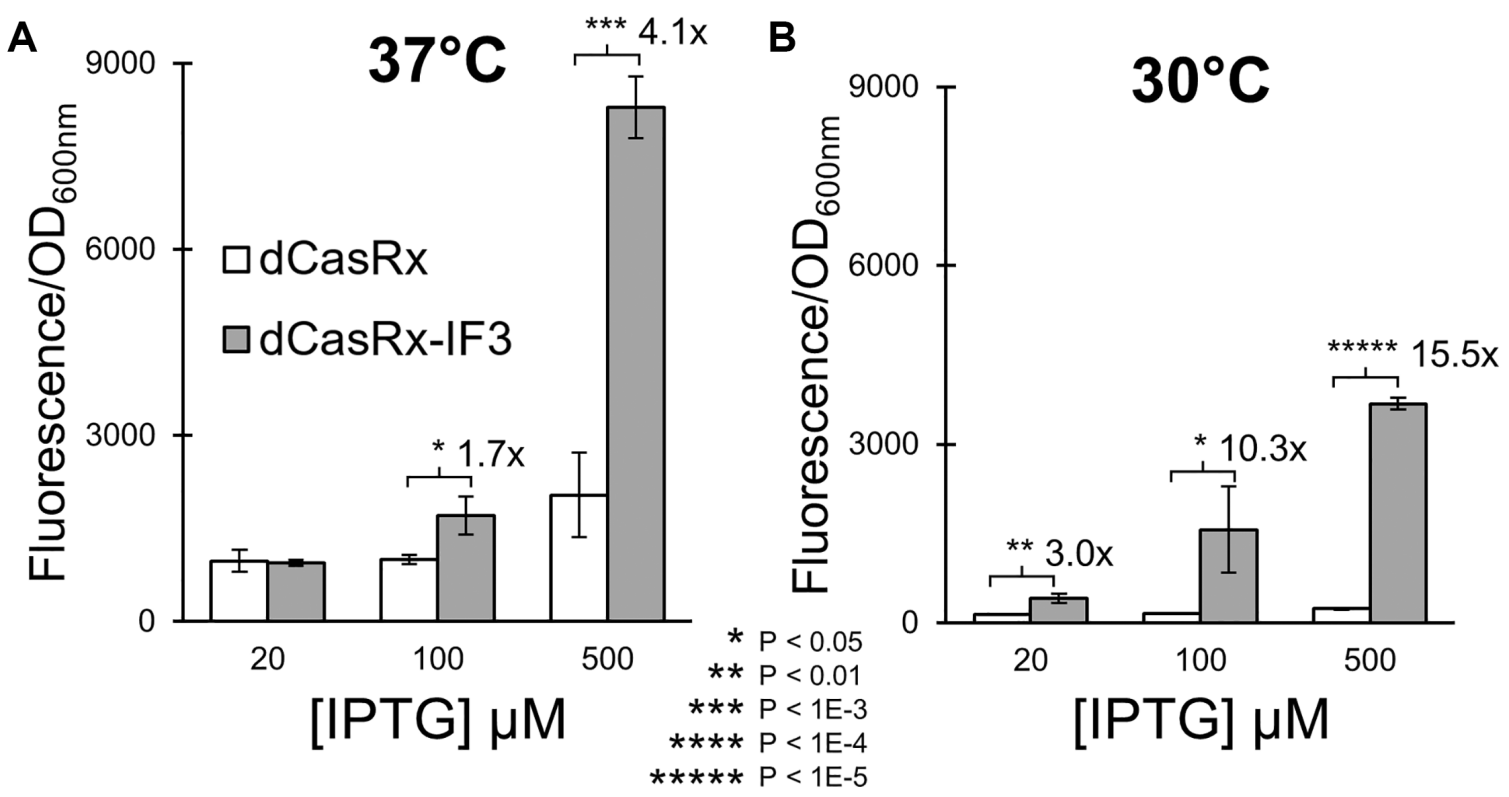
Increasing [IPTG] and reducing temperature enhances IF3-specific effect. A schematic of the revised experimental setup is presented in Supplementary Figure S6. (**A**) Normalized fluorescence of strains harboring either dCasRx (white bars) or dCasRx-IF3 (grey bars) alongside the RFP-1 20nt crRNA, when the experiment was performed at 37°C. (**B**) The same experiment, but performed at 30°C. All error bars represent standard deviation of biological triplicates. Asterisks indicate *P*-values of two-tailed type II Student's *t*-tests.

**Figure 4.**
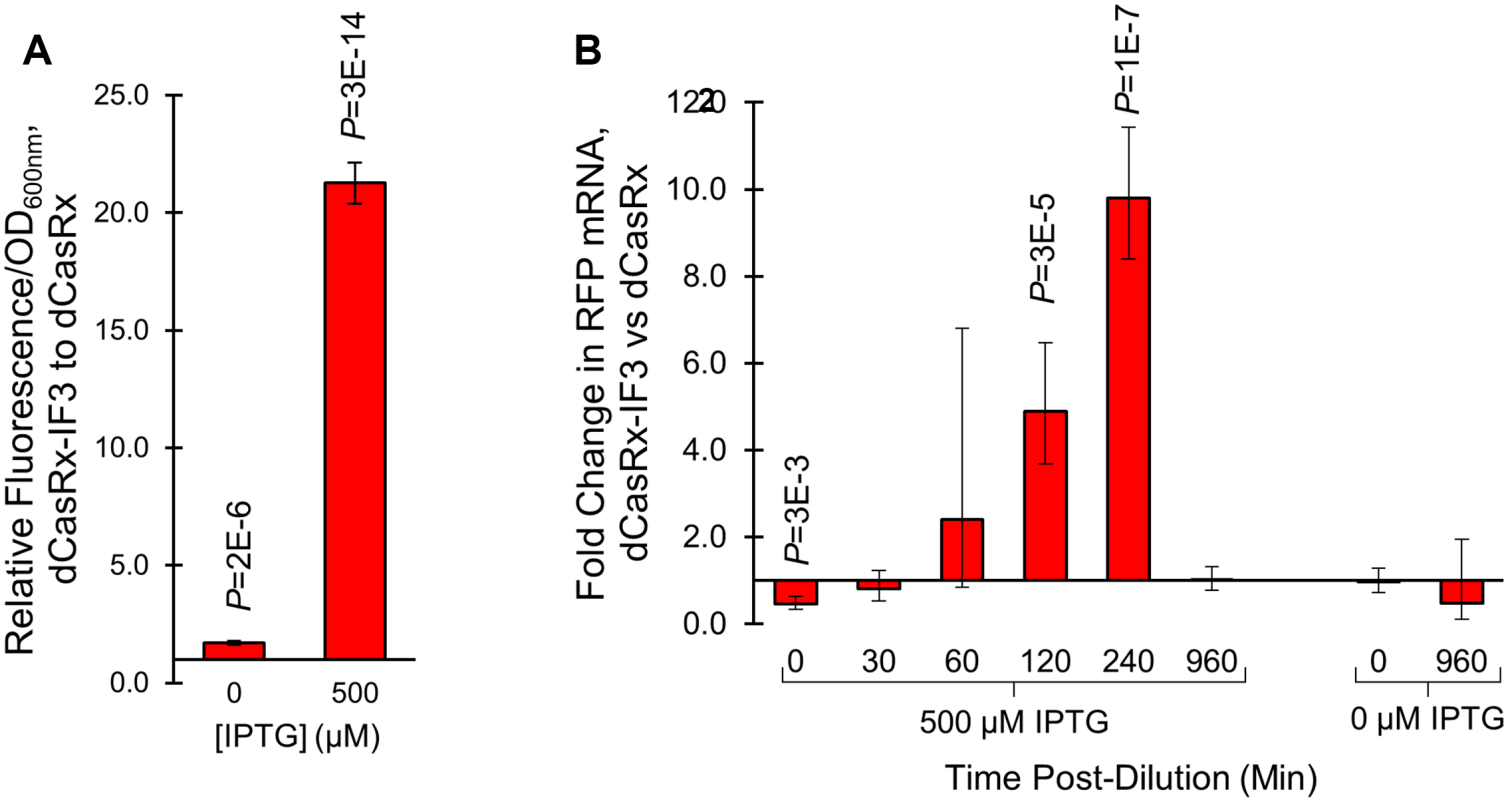
Influence of CRISPR-RNAa on RNA stability over time. (**A**) Fluorescence of dCasRx-IF3 strains relative to dCasRx strains when targeting *rfp* mRNA with crRNA RFP-1, using the experimental setup outlined in Supplementary Figure S7 (with volume increased to 1 ml to ensure adequate cell count for measuring RNA). Fluorescence was measured at 960 min in the final cultures, with pre-culturing induction using either 500 μM or 0 μM IPTG induction of *rfp* mRNA transcription. (**B**) RT-qPCR measurement of *rfp* mRNA in dCasRx-IF3 strains relative to dCasRx strains, quantified at the noted time post-dilution (Supplementary Figure S7 step 4). Samples were either induced with 500 μM IPTG (left) or IPTG was excluded (right) during pre-culture. All error bars represent standard deviations of four biological replicates. *P*-values were calculated using two-tailed type II Student's *t*-tests.

**Figure 5.**
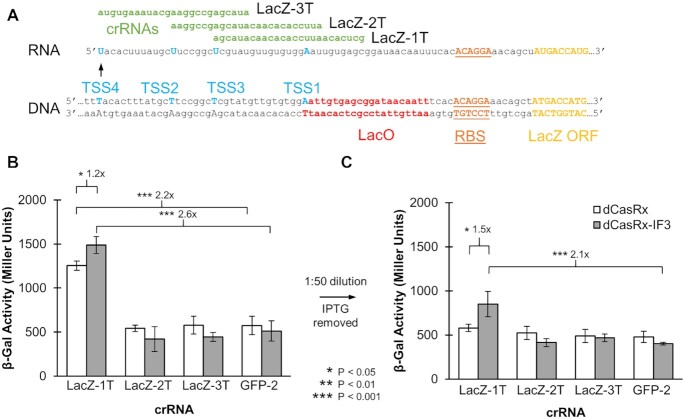
Expanding CRISPR-RNAa to enhance translation of the native *E. coli* gene *lacZ*, encoding β-galactosidase. (**A**) Target location of three crRNAs to enhance *lacZ* translation. Established transcription start sites (TSSs) are indicated, ranked in order of most common (TSS1) to least common (TSS4). (**B**) LacZ activity as measured using a β-galactosidase (‘Miller’) assay, performed at 30°C with 500 μM IPTG induction (as in Supplementary Figure S7, excluding step 4). (**C**) The same experiment, including step 4 (dilution and removal of IPTG) to enhance differences between dCasRx and dCasRx-IF3. All error bars represent standard deviation of biological triplicates. Asterisks indicate *P*-values of two-tailed type II Student's *t*-tests.

All strains were cultured in Luria-Bertani (LB) broth at 37°C supplemented with the appropriate antibiotics to maintain plasmid selection, unless otherwise stated. For cloning, all strains were selected on LB agar plates supplemented with the appropriate antibiotic at 37°C. Before all experiments, strains were streaked onto LB agar plates supplemented with the appropriate antibiotic and grown at 37°C to begin experiments from individual colonies. Bacteria were inoculated from individual colonies into 500 μl cultures in 96-well deep-well plates covered by a breathable seal, and grown at 37°C overnight. For Figures 1 and 2, Supplementary Figures S2, and S4, cultures were diluted 1:100 in fresh media excluding IPTG, and grown overnight again at 37°C. For kinetic experiments in Figure 2D, E, and Supplementary Figure S2B, growth was performed in 200 μl reactions in 96-well plates in a Synergy™ H1 Microplate Reader (BioTek) set to 37°C and continuous orbital shaking. For Figures 3–5, and Supplementary Figures S7–S13, cultures were diluted 1:100 in fresh media, grown for 10 h at 30°C or 37°C as indicated, diluted again 1:50 in fresh media, and grown overnight at 30°C or 37°C as indicated in Supplementary Figure S6. For Figure 4, volumes were increased in the 1:50 dilution from 500 μl to 1000 μl to ensure enough cells were collected for subsequent RNA analysis. Fluorescence and optical density measurements were then taken. For Figure 5B, the 1:100 dilution culture used to inoculate the 1:50 dilution was kept growing at 30°C and end measurements were also performed. For Supplementary Figure S11, the final 1:50 dilutions were also grown with kinetic measurements in 100 μl cultures as outlined above.

Carbenicillin (100 μg/ml) was used to maintain selection of crRNA plasmids, while kanamycin (50 μg/ml) was used to maintain selection of dCasRx plasmids. For induction of dCasRx variants, anhydrotetracycline (aTc) was utilized, at a concentration of 20 ng/ml for Figures 3 and 5, Supplementary Figures S3 and S7–S13 or at a concentration of 500 ng/ml for Figures 1 and 2, Supplementary Figures S4, and S5. For all other figures, aTc concentrations are listed within the figure. For induction of RFP and β-galactosidase, isopropyl β-d-1-thiogalactopyranoside (IPTG) was utilized, at a concentration of 100 μM for Figures 1 and 2, Supplementary Figures S4 and S5, or at a concentration of 500 μM for Figures 4 and 5, Supplementary Figures S3 and S7–S12. For all other figures, IPTG concentrations are listed within the figure. Unless otherwise noted, inducer was included at the time of culture inoculation or dilution.

For experiments in Figure 2 and Supplementary Figure S5A, only carbenicillin antibiotic selection was included for all cultures, while in Supplementary Figure S3 no antibiotics were included, to facilitate comparisons between strains with and without plasmid variants.

For Figure 4, five identical technical replicates 1 mL cultures were inoculated in the final 1:50 dilution as outlined in Supplementary Figure S6 step 5. One of each was used to collect RNA at the indicated timepoints, while the pre-dilution culture was used to collect RNA for time 0. For collecting RNA, cultures were immediately transferred into 1.5 ml Eppendorf tubes chilled on ice, and moved to a 4°C cold room. Samples were centrifuged at maximum speed for 2 min, the supernatant decanted, the samples spun again briefly, and the remaining supernatant removed with a pipette. Samples were then flash frozen in a dry ice-ethanol bath, and kept at –80°C for <48 h before all samples were processed simultaneously for RNA recovery.

### Plasmid construction

A list of plasmids used in this study are included in Supplementary Table S2, and are available through Addgene (https://www.addgene.org) or the authors upon reasonable request. A list of primers used for plasmid construction and validation are included in Supplementary Table S3. Plasmids encoding dCasRx (pEJC 1.5 CasRx) and a base crRNA cloning vector (pEJC 2.5.1 empty) were built in a previous publication (34), and used to construct plasmids used in this study. Codon-optimized sequences of native *infA* and *infC* (excluding the methionine start codon) were ordered as gBlocks (IDT) and used for insertion onto the C-terminus of dCasRx, linked by a flexible 3x(GGGGS) linker domain. Four to five DNA fragments were generated via PCR using primers PBO.ACT001 to PBO.ACT012, and Gibson Assembly was used to stitch these fragments together using the NEBuilder^®^ HiFi DNA Assembly Cloning Kit (New England BioLabs). Because the *E. coli* strains for expressing *rfp* contained chloramphenicol resistance, the chloramphenicol resistance marker on plasmid pEJC 1.5 was replaced with kanamycin resistance using plasmid pGI132 from our previous publication (43). This created plasmids pPBO.ACT001, pPBO.ACT002 and pPBO.ACT003 for expressing dCasRx, dCasRx-IF1 and dCasRx-IF3 respectively. We also used primers PBO.ACT023-24 to remove dCasRx from plasmid pPBO.ACT003, to create plasmid pPBO.ACT042 expressing aTc-inducible IF3.

A base plasmid for cloning constitutively expressed crRNA guides, PBO.ACT004 was created from pEJC 2.5.1 using primers PBO.ACT013 to PBO.ACT016. We noted the predicted crRNAs contained a substantial extended 3′ fragment (see Supplementary Figure S4) (44). We thus created plasmid PBO.ACT005, expressing a crRNA with a significantly shorter 3′ fragment predicted for the crRNAs, using primers PBO.ACT015 to PBO.ACT018. To facilitate cloning of new guide targets, we also created PBO.ACT006 containing eGFP (enhanced Green Fluorescent Protein) at the target crRNA cloning site using primers PBO.ACT014, PBO.ACT015 and PBO.ACT019 to PBO.ACT022. For creating new crRNA target plasmids, oligos were ordered and duplexed as previously described (45) and assembled with PBO.ACT004-PBO.ACT006 using BsmBI Golden Gate Assembly. This created plasmids PBO.ACT007-041 for constitutively expressing crRNAs guides. We also used primers PBO.ACT025-30 to convert pPBO.ACT012 into plasmid pPBO.ACT043 with crystal-violet inducible crRNAs with target RFP-1 (46). Sequencing primers used to verify all constructs, PBO.SEQ001-009, are listed in Supplementary Table S3.

### Growth and fluorescence measurements

Optical density (OD) and fluorescence measurements were performed using a Synergy™ H1 Microplate Reader (BioTek). Endpoint fluorescence was measured using 590nm excitation and 635nm emission, and normalized to optical density 600 nm measurements where noted. For kinetic experiments, measurements were performed at 20-min intervals.

### Flow cytometry

Flow cytometry experiments were performed using an autosampler-equipped Accuri™ C6 Plus Flow Cytometer system (BD Biosciences). The four biological replicates collected in Supplementary Figure S8A were used for flow cytometry analysis. 90 μl of culture was transferred to 10 μl of 37% formaldehyde solution (Sigma-Aldrich) and incubated at room temperature for 15 min, after which 20 μl of samples were diluted into 180 μl of phosphate-buffered saline. Samples were then analyzed on the Accuri system, with a minimum of ∼500 000 ungated events were collected. Data was analyzed using FlowJo^®^ version 10.8.1.

### RNA extraction and reverse transcription

From the same four biological replicates that were used to measure fluorescence in Supplementary Figure S8A, 300 μl were collected at the same time point and immediately processed. For the same four biological replicates that were used to measure fluorescence in Figure 4A, the five separate 1 ml technical replicate cultures were collected at each noted timepoint (as well as starting culture at time 0) and immediately frozen, for subsequent processing. These samples were thawed on ice for subsequent RNA collection within 48 h. RNA was collected using the GeneJET RNA Purification Kit (Thermo Scientific™) supplemented with lysozyme (Thermo Scientific™), following the manufacturer's instructions for bacterial total RNA purification. DNase I (Thermo Scientific™) treatment was subsequently used to remove genomic DNA following the manufacturer's instructions, with a final RNA concentration of approximately 5, 23 or 500 ng/μl. DNase I was diluted as appropriate to ensure no more than 1 unit per μg of input RNA, as recommended by the manufacturer. RNA was then converted into cDNA using the RevertAid First Strand cDNA Synthesis Kit (Thermo Scientific™) following the manufacturer's instructions for 10 μl reactions containing 0.5 μl random hexamer mix, 2 μl 5× reaction buffer, 0.5 μl RiboLock, 1 μl 10 mM dNTP and 0.5 μl Reverse Transcriptase. Samples were heated at 25°C for 5 min, followed by 42°C incubation for 60 min and heat inactivation at 70°C for 5 min. cDNA was diluted to approximately 2.5 or 5.0 (Figure 4) or 6.25 ng/μl (Supplementary Figure S8), stored at –80°C for less than 2 months and used for downstream qPCR analysis. RNA and DNA quality and quantity were assessed using a NanoDrop™ 2000 (Thermo Scientific™).

### Genomic DNA extraction

From the same four biological replicates that were used to measure fluorescence in Supplementary Figure S8C, 200 μl of culture was collected at the same time point for extracting genomic DNA for quantification in Supplementary Figure S8D. The Wizard^®^ Genomic DNA Purification Kit (Promega) was used to extract genomic DNA, following the manufacturer's instructions for Gram-negative bacteria. Background RNA was removed using the manufacturer's recommended 20 min RNase A incubation. gDNA concentrations were adjusted to 8 ng/μl, and used immediately for downstream qPCR analysis.

### Quantitative PCR

Quantitative Real-Time PCR (qPCR) was performed in accordance with the *M*inimum *I*nformation for Publication of *Q*uantitative Real-Time PCR *E*xperiments (MIQE). Relative nucleic acid concentrations were quantified using PowerUp™ SYBR™ Green 2x Universal Master Mix (Applied Biosystems™), using the manufacturer's recommendations with no modifications or supplements. qPCR was performed manually in-house on a CFX96 or CFX384 Real-Time PCR Detection System (BioRad). For all samples, 10 μl technical duplicate reactions were performed containing 2.5 or 5.0 cDNA (for Figure 4, with 5.0 ng for time points 30, 60, and 120 min and 2.5 ng for the remaining time points), or 20 μl technical duplicate reactions containing 13 ng cDNA or 16 ng gDNA (for Supplementary Figure S8). Copy numbers of *rfp* were normalized to the housekeeping gene, *hcaT*, previously shown to be a good reference gene for *E. coli* (47). 500 nM unmodified primers produced with standard purification (IDT) were used, and primers are listed in Supplementary Table S3. Primers were designed based on the *E. coli* DH1 genome (Accession Number AP012030) with BLAST analysis to determine negligible off-targets. Amplicon lengths are 114 and 90 bp for *rfp* and *hcaT*, respectively. Reactions were performed with an initial 50°C 2-min incubation for uracil–DNA glycosylase activation, followed by a 95°C 2-min incubation for polymerase activation. Forty amplification cycles were subsequently performed using a 95°C 15 s denaturation step and 60°C 1-min annealing/extension step. CFX manager software (version 3.1) was used to calculate instrument-reported CT values of technical duplicates were averaged and used to calculate 2^–ΔCT^ and 2^–ΔΔCT^ values of *rfp* copy number relative to *hcaT*. DNA contamination was assessed using a no reverse transcriptase control, as well as a no cDNA control for each primer pair. A melt curve was performed after each amplification, and a single melt curve peak (around 82.5°C and 83.5°C for *rfp* and *hcaT*, respectively) was used to verify primer specificity. Primer efficiencies were calculated as 98.3% (standard curve slope = –3.4, intercept = 16.5, *R*^2^ = 0.97) and 107.8% (standard curve slope = –3.1, intercept = 24.5, *R*^2^ = 0.96) for *rfp* and *hcaT* primers respectively. No CT was observed for the NTC with *hcaT* primers, while the CT for the NTC with *rfp* primers was 35. Neither the linear dynamic range nor limit of detection were explored, nor was the intra-assay variation. No outliers were removed. Statistical significance was calculated using Student's two-tailed type II *t-*test comparing ΔCT values between strains harboring dCasRx and dCasRx-IF3 with the noted crRNA.

### β-Galactosidase assay

Measurement of LacZ activity via the β-Galactosidase (also known as Miller) assay was performed as outlined previously (48). Measurements were performed using a Synergy™ H1 Microplate Reader (BioTek).

## RESULTS

### Fusion of translation initiation factors to dCasRx enables targeted translational activation

For exploring targeted activation of translation, we utilized a catalytically deactivated variant of *Ruminococcus flavefaciens* XPD30002 Cas13d, dubbed ‘dCasRx’ (31). dCasRx is a Cas13 ortholog that does not possess target sequence constraints due to its lack of a protospacer flanking sequence (PFS) requirement. We cloned onto the C-terminus a flexible 3x(GGGGS) protein linker domain, followed by genes *infA* or *infC* encoding *E. coli* translation initiation factors IF1 and IF3 respectively. These fusions replaced an original 2× FLAG-tag at the protein's C-terminus in its original design, which itself was used as a control for comparison of translational activation. As a target for gene activation, we utilized a strain of *E. coli* with ∼66 copies of *rfp* integrated into its genome at the *intA* loci under control of the IPTG-inducible LacUV5 promoter (42). A guide crRNA was constructed to target the first 20 nts of *rfp* mRNA, exactly 46–26 nts upstream of the ribosomal binding site (RBS) (Figure 1A).

We moved on to test the impact these constructs had on fluorescence by co-transforming each alongside *rfp*-targeting crRNAs. Our initial attempts yielded small (1.3-fold) yet statistically significant (*P* = 0.02) increases in fluorescence of dCasRx-IF3 strains relative to the dCasRx control strains (Supplementary Figure S1). We saw no substantial impact on growth (Supplementary Figure S2). We found that tuning the induction conditions of the experiment substantially influenced these differences. Our most optimal experimental setup for differentiating dCasRx from the dCasRx-IF fusions involved first culturing samples overnight in the presence of both aTc and IPTG, followed by a 1:100 dilution of cultures into media containing aTc but no IPTG, thus abrogating production of target *rfp* mRNA (Supplementary Figure S1E).

With these tuned experimental conditions, we found strains expressing *rfp*-targeting crRNAs and harboring either dCasRx-IF1 or dCasRx-IF3 to fluoresce significantly brighter than strains harboring dCasRx (Figure 1B). dCasRx-IF1 enhanced fluorescence 2.2 ± 0.2 fold (*P* = 3E–4) and dCasRx-IF3 enhanced fluorescence 3.3 ± 0.5 fold (*P* = 1E–3) respectively, relative to dCasRx strains. These fluorescence differences are visually distinct, and observable to the human eye (Figure 1C). We confirmed that overexpression of IF3 alone in the absence of dCasRx did not result in this substantial increase in fluorescence (Supplementary Figure S3). As the fusion to IF3 consistently resulted in the highest levels of fluorescence, we chose to focus on comparisons between dCasRx-IF3 and dCasRx for subsequent experiments.

### Fusion of IF3 to dCasRx provides maximal expression enhancement, and the degree of enhancement is dependent on target-location on mRNA

As small RNAs have been shown to enhance protein expression encoded by a complementary strand of RNA under the right conditions (49,50), we next sought to demonstrate that the guide crRNAs alone were not responsible for these results. Additionally, as researchers have shown that CRISPR tools for activating transcription are heavily influenced by the target location of crRNA guides (10), we wanted to systematically explore a library of different target locations to determine good design principles for using CRISPR-RNAa. For this, we compared the fluorescence of strains harboring only the crRNA guides with those harboring both crRNAs and either dCasRx or dCasRx-IF3. We first explored varying the length of the crRNA from our original 20 nt construct (RFP-1) to 15–25 nts (RFP-2 to RFP-11) (Figure 2A). Two variants of a crRNA targeting *gfp* were used as controls, both demonstrative of basal fluorescence levels. Negligible fluorescence differences were observed between strains targeting these *gfp* controls and harboring either crRNA alone, in the presence of dCasRx, or dCasRx-IF3. The latter *gfp-*targeting crRNA (GFP-2) was used for comparison against other strains.

All crRNA variants of RFP-1 to RFP-11 were able to increase expression of *rfp* alone, without coupling with dCasRx or dCasRx-IF3. The fold-level of activation of these crRNAs was between 1.5 ± 0.1 (RFP-6, *P* = 1E–3) and 3.6 ± 0.7 (RFP-7, *P* = 2E–3). Taken together, these crRNA-only results demonstrate that trans-activating RNA–RNA interactions played a significant role in modulating RFP expression.

Notably, introduction of dCasRx into crRNA-expressing strains substantially enhanced fluorescence beyond any enhancement observed by crRNAs alone (Figure 2A). There were no substantial differences with the two shortest crRNAs (RFP-2 and RFP-3) with and without dCasRx proteins. However, with a length of 17nts (RFP-4) the effect of addition of dCasRx (2.1 ± 0.6, *P* = 0.03) and dCasRx-IF3 (2.0 ± 0.2, *P* = 8E–5) relative to the crRNA alone strain emerged. Increasing the crRNA target length further enhanced the activation effect of both proteins, in particular for dCasRx-IF3. With crRNA lengths of 19 nts and above, the fold increase of fluorescence of strains harboring dCasRx-IF3 was statistically greater than strains harboring dCasRx (excluding RFP-8 crRNA), ranging between 1.3 ± 0.1, (RFP-7 *P* = 4E–3) to 2.0 ± 0.2 (RFP-10, *P* = 0.02). For both proteins, the 23nt RFP-9 crRNA appeared to provide maximum gene expression activation, relative to GFP-2 crRNA (7.6 ± 0.6, *P* = 2E–5 and 12.5 ± 0.5, *P* = 4E–7 for dCasRx and dCasRx-IF3, respectively).

These results indicate that dCasRx interaction with the mRNA provides some mechanism for enhancing fluorescent protein levels independent of RNA–RNA effects or IF3 effects alone (potentially by providing protection from RNase E, see Discussion for details). The addition of IF3 to dCasRx appears to provide some additional benefit to enhancing gene expression, possibly by increasing translation initiation as we intended or mRNA stabilization (see Discussion).

Next, we explored the effect of varying the target crRNA location across the mRNA (Figure 2B). Strikingly, shifting the crRNA target from the start of the 5′ UTR to just 10 nts downstream (RFP-12) removed virtually all activation activity. Progressing any further towards the RBS resulted in repression of RFP relative to GFP-targeting crRNAs, regardless of if the crRNA was alone (0.78 ± 0.06, *P* = 7E–3), coupled with dCasRx (0.71 ± 0.03, *P* = 4E–4), or coupled with dCasRx-IF3 (0.51 ± 0.06, *P* = 1E–4). Maximum repression was obtained when targeting directly to the RBS (RFP-15), again with the crRNA alone (0.69 ± 0.10, *P* = 8E–3), coupled with dCasRx (0.38 ± 0.11, *P* = 8E–4), or coupled with dCasRx-IF3 (0.27 ± 0.03, *P* = 4E–6). A slight inhibitory effect was also seen when targeting with the start of the ORF (RFP-16). No effect was observed when targeting in the middle of the ORF (330–350nts from the translation start site, RFP-17) or when targeting the 3′ UTR (RFP-18). These results suggest that, despite being designed for translational activation, dCasRx-IF3 efficiently represses translation when occluding the RBS and could therefore be employed in multiplexing strategies for simultaneous knockdown and overexpression.

At this point, we recognized that our crRNAs used a sub-optimal transcription termination sequence, leading to a 300nt 3′ overhang between the end of our crRNAs and the actual transcription termination (Supplementary Figure S4A). To ensure this did not influence our results, we redesigned our crRNA base-cloning construct to remove this overhang. The names of all crRNAs using this shortened terminator are annotated as ending in ‘T’ to indicate this change. We explored 3 of our previous crRNAs (RFP-1, RFP-8 and RFP-10) with and without the terminator change. We found the effect was either negligible, or slightly improved activation efficiency (particularly of the crRNA alone) (Supplementary Figure S4B). Our highest fold-activation of dCasRx-IF3 relative to the GFP-2 targeting crRNA control (13.7 ± 0.6, *P* = 4E–7) was obtained using the RFP-8T crRNA, indicating that use of these shorter terminators is preferable.

We therefore proceeded to explore our next question with crRNAs using this shorter terminator. We wanted to systematically explore the space between RFP-1 and RFP-12 step-by-step, using crRNAs RFP-19T to RFP-27T respectively (Figure 2C). We found that stepping 2 nts downstream from the start of the 5′ UTR slightly increased dCasRx-IF3 activation relative to GFP-2 dCasRx-IF3 strains to 11.9 ± 0.4 fold (*P* = 1E–8). A large drop in activation was then seen when transitioning further downstream, with the exception of RFP-23T targeting 5 nts downstream of the 5′ UTR start where both dCasRx and dCasRx-IF3 still maintained substantial activation potential (7.5 ± 2.5, *P* = 0.01 and 7.6 ± 0.4, *P* = 7E–6 for dCasRx and dCasRx-IF3 respectively).

We repeated this experiment with a subset of crRNAs alone, and in combination with dCasRx and dCasRx-IF3 (Supplementary Figure S5). These results largely confirmed the reproducibility of the results demonstrated in Figure 2A-C. In one of these experiments, we were able to obtain a slightly higher fold-activation of dCasRx-IF3 relative to the GFP-2 targeting crRNA control (16.0 ± 1.5 fold, *P* = 9.6E–6) when using RFP-11 (Supplementary Figure S5A). This is comparable to the 23-fold activation first reported with transcription-based CRISPRa in bacteria, which required deletion of native *rpoZ* to achieve (10).

Finally, we also performed kinetic experiments in parallel to provide an insight into the dynamics of fluorescence changes over time, using our longest crRNA RFP-11. All strains exhibited nearly identical growth profiles (Figure 2D). While there were no significant differences (*P* = 0.13) in growth rates between dCasRx (0.42 ± 0.04 h^–1^) and dCasRx-IF3 (0.36 ± 0.05 h^–1^) strains, there was a slight yet noticeable increase in the lag time of dCasRx-IF3 strains relative to dCasRx strains. This suggests that dCasRx-IF3 does introduce a small influence in the shift into exponential growth. Fluorescence of these strains remained relatively similar in early growth, yet diverged markedly towards later growth (Figure 2E).

### Enhancing the IF3-specific activation of CRISPR-RNAa

While the results above show that dCasRx-IF3 enhances expression more than dCasRx alone, the greatest difference observed was only 3.3-fold. We therefore sought to determine parameters which increase the IF3-specific effect of CRISPR-RNAa. We reduced the abundance of dCasRx and dCasRx-IF3 molecules by reducing aTc induction concentration from 500 to 20 ng/μl, to ensure the Cas proteins were not saturating the target mRNAs. We also delayed induction of the target *rfp* mRNA production from the start of the pre-culture, to 5 h after inoculation, a common strategy for enhancing expression of difficult proteins (51).

Using these slightly altered experimental parameters (as outlined in Supplementary Figure S6), we explored varying the concentration of IPTG induction. We first performed this experiment at 37°C (Figure 3A). Reducing IPTG concentration to 20 μM eliminated differences in fluorescence between these strains (*P* = 0.21), while increasing IPTG concentration increased the fold change in fluorescence activation from 1.7 ± 0.3-fold (*P* = 0.02) to 4.1 ± 1.4-fold (*P* = 2E–4). This indicates a dose-dependent response tied to the copy number of *rfp* mRNAs.

Next, we repeated the experiment at 30°C (Figure 3B). Low temperatures are frequently used to slow protein production rates, providing time for proper folding and to discourage protein aggregation (51). We found that this lower temperature further accentuated the differences between dCasRx and dCasRx-IF3. The fold difference between dCasRx-IF3 and dCasRx was 3.0 ± 0.6 (*P* = 5E–3) at the lowest IPTG concentration, 10.3 ± 4.8 (*P* = 0.03) at the intermediate IPTG concentration, and 15.5 ± 1.0 (*P* = 5E–7) at the highest IPTG concentration.

These trends were largely recapitulated when the experiment was performed with IPTG induction included at the start of the experiment, rather than after 5 h of growth (Supplementary Figure S6). In this case, lower IPTG concentrations resulted in less fluorescence differences overall between dCasRx and dCasRx-IF3. We also explored the 22nt crRNA variant RFP-8T using this new experimental setup. (Supplementary Figure S7). The trends presented in Figure 3 were largely recapitulated with this slightly altered guide RNA; at 30°C, the fold difference between dCasRx-IF3 and dCasRx was 2.1 ± 1.1 (*P* = 0.03) at the lowest IPTG concentration, 6.9 ± 1.6 (*P* = 0.003) at the intermediate IPTG concentration, and 9.3 ± 7.2 (*P* = 2E–3) at the highest IPTG concentration.

### Quantifying copy number differences

The reporter *E. coli* strain used in this work encodes multiple copies of *rfp* integrated into its genome. This strain was built specifically to maintain stable copy number, and it has been reported to maintain 71% of its original copies of *rfp* after a billion-fold outgrowth in the absence of antibiotics (42). However, to ensure that copy number variations emerging during strain construction did not lead to the results presented here, we explored the copy number of *rfp* at both the RNA and DNA level.

We first quantified target RNA abundance in the strains at the time of fluorescence measurements, using the protocol outlined in Supplementary Figure S6 (at 30°C) for strains harboring dCasRx variants and RFP-1 and RFP-8T crRNAs. Fold-fluorescence enhancement of dCasRx-IF3 relative to dCasRx strains was 7.2 ± 0.5 (*P* = 7E–8) and 3.9 ± 0.3 (*P* = 4E–7) when harboring either the 20 nt RFP-1 or 22 nt RFP-8T crRNA respectively (Supplementary Figure S8A). Using flow cytometry, we noted a uniform population distribution in both cell shape and fluorescence distribution, indicating no obvious emergence of sub-populations due to copy number loss during the experiment (Supplementary Figure S9 and Supplementary Table S4). Using RT-qPCR, we found that both dCasRx-IF3 strains harbored more copies of *rfp* mRNA than their corresponding dCasRx strains. Specifically, *rfp* mRNA abundance was 2.0 ± 0.4 (*P* = 0.001) and 4.5 ± 1.9 (*P* = 4E–4) fold higher in the dCasRx-IF3 strains relative to the dCasRx strains harboring either the 20 nt RFP-1 or 22 nt RFP-8T crRNA, respectively (Supplementary Figure S8B).

We next measured *rfp* copy number at the DNA level to determine if potential copy number changes might contribute to increased *rfp* transcript abundance. We repeated the experiment, this time including the GFP-2 control. Fold-enhancement of fluorescence in the case of dCasRx-IF3 relative to dCasRx strains was 7.7 ± 1.1 (*P* = 1E–9) and 8.7 ± 4.6 (*P* = 7E–7) when harboring either the 20 nt RFP-1 or 22 nt RFP-8T crRNA, respectively (Supplementary Figure S8C). There was a slight drop in fluorescence for strains harboring dCasRx-IF3 relative to dCasRx when harboring the GFP-targeting crRNA (fold change = 0.89 ± 0.05, *P* = 4E–3), possibly due to the lag time effect noted earlier. We found no difference in the copy number of *rfp* in the 20 nt RFP-1 strains, where the relative *rfp* copy number of dCasRx-IF3 to dCasRx was 1.0 ± 0.2 (*P* = 0.29) (Supplementary Figure S8D). However, for both of the other strains, there was a slight increase in the copy number of the dCasRx-IF3 relative to the dCasRx strains. The relative *rfp* copy number of dCasRx-IF3 to dCasRx was 1.6 ± 0.3 (*P* = 5E–3) when harboring the 22 nt RFP-8T crRNA, and 1.9 ± 0.5 (*P* = 5E–3) when harboring the GFP-targeting crRNA. Notably, while strains of dCasRx-IF3 with these two crRNAs exhibited slightly higher copy numbers at the DNA level, only in targeting *rfp* did this result in enhanced fluorescence. These results suggest that while copy number differences were indeed possible at both the DNA and RNA level, they do not appear to have substantially influenced the enhanced mRNA copy numbers.

We next revisited the differences in mRNA copy numbers, this time focusing on quantifying relative RNA copy numbers over time between dCasRx and dCasRx-IF3 strains both harboring RFP-1 crRNA targets (Figure 4). We repeated the experiment as outlined in Figure 3A, except this time we specifically fixed the endpoint measurement to 16 h, while also collecting timepoints at 0, 30, 60, 120 and 240 min post-dilution (IE after Supplementary Figure S6, step 4). We also included controls with no IPTG induction at any point, to ensure leaky expression was not the cause of these differences. We measured endpoint fluorescence and optical densities of these cultures at 960 min (Supplementary Figure S10). We also setup a kinetic experiment in parallel to capture growth and fluorescence profiles over time of these cultures (Supplementary Figure S11). We found that the relative normalized fluorescence of dCasRx-IF3 to dCasRx strains without IPTG induction was 1.7 ± 0.1 (*P* = 2E–6), indicating a slight effect of leaky expression through the IPTG-inducible promoter (Figure 4A). However, we found a much greater difference when IPTG was included 21.3 ± 0.9 (*P* = 3E–14), indicating this was not the sole reason for differences in these strains.

We next explored the copy numbers of *rfp* mRNA over time in these conditions. At the time of culture dilution, we found that there were roughly half as many copies of *rfp* mRNA in the IPTG-induced dCasRx-IF3 strains relative to dCasRx strains (0.46-fold, *P* = 3E–3). There were no differences in *rfp* mRNA copy number in the uninduced cultures. Within the first 60 min of culture dilution, we could find no difference in copy number. However, by 120 min, we found 4.9-fold more *rfp* mRNA in the dCasRx-IF3 strain (*P* = 3E–5). By 240 min, this fold-difference was increased to 9.8 (*P* = 1E–7). By the end of the experiment, the relative *rfp* copy numbers had normalized between these strains, both with and without IPTG induction. Raw CT values are presented in Supplementary Figure S12.

Collectively, these results suggest that *rfp* mRNA was more stable in dCasRx-IF3 strains for at least the first 4 h post-culture dilution. This could be due to an increase in translation initiation caused by IF3 leading to greater ribosome saturation of the mRNA, thereby extending mRNA half-life. Indeed, increasing translation initiation by varying the strength of the RBS has been shown to not only increase gene expression, but copy number of the target mRNA up to 11.8-fold (52), lending credence to this hypothesis.

### Activation of native β-galactosidase

To further validate the application of dCasRx-IF3 for enhancing gene expression, we explored activation of an endogenous gene. We turned to the well-characterized *lacZ* gene of *E. coli*, encoding β-galactosidase. While the transcription start site (TSS) of *lacZ* is canonically at the beginning of the LacO binding region, transcription can also begin at three other locations (numbered in order of decreasing strength from TSS1 to TSS4) (53,54). We designed three 25 nt crRNAs (with the aforementioned shortened terminator sequence) to target the 5′UTR of *lacZ*, corresponding to these three less-frequent TSSs (Figure 5A). TSS1 was not targeted, as this would encroach onto the RBS (25 bp upstream), as evidenced by our previous results with crRNA RFP-13 (26 bp upstream). We transformed these constructs into AG1, a base strain of DH1 *E. coli* with the native operon of *lacZ* kept intact. Experiments were conducted using the protocol developed in Supplementary Figure S6, with the temperature maintained at 30°C and 500 μM IPTG used to induce the *lac* operon. A β-Galactosidase assay was performed to quantify β-galactosidase expression both before and after dilution and removal of IPTG (48).

Before dilution, strains harboring the LacZ1 crRNA alongside both dCasRx and dCasRx-IF3 enhanced β-galactosidase activity (Figure 5B). The relative Miller units between strains harboring dCasRx and either the LacZ1 or control GFP-2 crRNAs was 2.2 ± 0.4 (*P* = 5E–4). Likewise, the relative Miller units between strains harboring dCasRx and either the LacZ1 or control GFP crRNAs was 2.6 ± 0.5 (*P* = 4E–4). Strains harboring dCasRx-IF3 and the LacZ1 crRNA showed a 1.2 ± 0.1 (*P* = 0.02) fold increase in β-galactosidase activity relative to strains harboring dCasRx and the LacZ1 crRNA. There were no significant differences in any of the remaining strains.

Samples were diluted 1:50 and IPTG was removed, and β-galactosidase activity was again measured at the end of growth. While the strains harboring LacZ1 crRNAs again produced the most Miller units, there was no significant difference between the strain harboring dCasRx and LacZ1 crRNA and the strain harboring dCasRx and a GFP crRNA (1.2 ± 0.2, *P* = 0.24). However, there was a significant enhancement between the strain harboring dCasRx-IF3 and LacZ1 and the strain harboring dCasRx-IF3 and a GFP crRNA (2.1 ± 0.4, *P* = 0.02). The difference between dCasRx and dCasRx-IF3 was also enhanced. The LacZ1 crRNA strain harboring dCasRx-IF3 produced 1.5 ± 0.3 (*P* = 0.03) fold more Miller units than that harboring dCasRx. Again, no significant differences were observed between any of the remaining strains. Collectively, these results further validate the application of CRISPRa for activating protein expression at the translational level.

## DISCUSSION

Even before researchers first co-opted CRISPR-Cas machinery for genetic manipulation, they pondered tethering of translation initiation factors to RNA-targeting proteins for translation efficiency enhancement (55). While scientists have proposed reconfiguring CRISPR machinery to enhance translation (56), it has not been demonstrated in practice (with the exception of CRISPR YTHDF1-mediated 1.4-fold activation in human HEK293T cells (57)), and efforts have largely been focused on controlling transcription.

Here we fill this conspicuous gap by developing CRISPR-RNAa, an approach in which RNA-targeting dCas13-IF fusions are targeted to 5′ UTRs to increase protein production by enhancing translation of the target mRNA-encoded gene. We first show that dCasRx-IF1 and dCasRx-IF3 induce stronger fluorescence than dCasRx when targeted to the start of the 5′UTR of *rfp* mRNA. We next decouple the individual effects of the guide crRNA, the binding of dCasRx, and IF3 itself. We show that crRNAs alone can slightly enhance gene expression (1.5–3.7-fold), likely through previously established RNA-RNA activating interactions (49,50). Addition of dCasRx increases this effect 2.4-fold. As RNase E, the primary enzyme responsible for mRNA degradation in *E. coli*, has a preference for 5′ monophosphorylated RNA (58–60), it is possible that dCasRx binding sterically hinders RNase E recognition of the 5′ region and consequently preserves the target mRNA’s half-life (61). This is supported by our observations in Figure 2 that shifting the target location of dCasRx 10 nt downstream of the 5′ UTR failed to activate (or inhibit) gene expression, and also the observation that activation decreased as we shifted the target location 1nt at a time downstream from the start of the 5′UTR.

We proceed to show that tethering translation initiation factors IF1 and IF3 to dCasRx further enhances gene expression, which we theorize is due to the IF’s recruitment of 30S ribosome subunits to the RBS of target mRNA-encoded genes. For evidence of this, we note the increased RFP expression of dCasRx-IF3 relative to dCasRx strains in Figure 2. This is further supported by our results in Figure 4, where *rfp* mRNA appears more stable in dCasRx-IF3 strains relative to dCasRx strains within the first 4 h of removal of IPTG induction. Enhanced translation initiation rates have been directly tied to higher mRNA levels due to protection of the mRNA by ribosomes from RNases, particularly through preventing RNase E endonucleolytic cleavage (52,62,63). Furthermore, researchers have argued that a competition between translation initiation rates and mRNA decay is the major determinant of mRNA half-life in yeast (64).

In a similar vein as RNA half-life, the influence of growth phase on CRISPR-RNAa is of interest. Free 70S ribosomes are prolific during exponential growth (upwards of 70 000 units) and are thought not to be rate-limiting to translation (65). However, during stationary phase, they can be reduced to as little as 2000 units as they transition into functionally inactive 100S dimers, promoting hibernation (66). This stationary-phase effect is accompanied by a similar reduction in IF3 levels (67) and an overall downregulation of translation initiation (68). Providing excess IF3 has been shown to help dissociate 100S ribosomes into functional 70S ribosomes (69). The influence of 100S ribosome formation and related hibernation factors such as RMF, HPF, and YfiA on CRISPR-RNAa therefore warrants further investigation (70).

Many components of our current implementation of CRISPR-RNAa could be optimized in the future. Here we explore only one form of protein linker, and only through tethering to the C-terminus. Varying the linker location, as well as its structure, could improve functionality. Further investigating the IF domains is also of interest. For instance, IF3 is a two-part protein consisting of an essential C-terminal domain (that performs the bulk of its functions) connected by a flexible hydrophilic linker to a non-essential N-terminal domain (71,72). Future iterations of CRISPR-RNAa could explore these domains individually to reduce size or improve efficiency.

The crRNA might also be made to be inducible, to provide further tunable control. We explore this briefly in Supplementary Figure S13 by placing the crRNA under control of a crystal-violet inducible promoter (46). However, this modification substantially extends the 5′ end of the crRNA and has strong secondary structure. The secondary structure of the 5′ stem has been shown to be critical for CasRx targeting efficacy (73). As many inducible promoters necessitate an element downstream of the +1 transcription start site, the fact that slight modifications to the crRNA 5′ stem can have large effects on CasRx activity suggests that detailed optimization may be required for obtaining the ideal crRNA structure with each different inducible promoter. Future iterations of CRISPR-RNAa using inducible guide RNAs could include elements such as the hammerhead ribozyme to tightly control the 5′ sequence of dCasRx crRNAs (74).

Of note, the target gene and its mRNA copy number might constrain the efficiency of translational activation. We saw significantly greater success of CRISPR-RNAa in activating a heterologous gene expressed at a high DNA copy number (*rfp*) than a native gene expressed from a single copy (*lacZ*). CRISPR-RNAa is an overexpression method, and factors peculiar to the target gene product that normally effect any overexpression experiment, such as folding rates or co-translational regulation, should still be expected to have an influence. Relatedly, we determined that lowering the temperature, a common tactic for slowing production rates to improve folding of heterologous proteins (51), enhanced the difference between dCasRx and dCasRx-IF3.

Expansion of CRISPR-RNAa into other organisms is also of interest. To this end, it is important to note the highly conserved nature of the components and processes of translation across all kingdoms of life (75). Within prokaryotes, IF3 is widely conserved, with a fascinatingly unique non-AUG start codon (40). Indeed, it is one of only two genes in *E. coli* (alongside the nonessential *pcnB*) to start translation from an AUU codon (76), which has been implicated to be a result of IF3’s capacity for self-regulation (77–79). IF3 discrimination against its own AUU start codon reduces its own translation initiation at high IF3 concentrations, which has been correlated with a reduction in *infC* coding mRNA in *B. subtills* (80).

While eukaryotes require additional IFs, forms of prokaryotic IF1, IF2 and IF3 are preserved in some form (81,82). The C-terminal domain of IF3 in particular bears similarity in both form and function to eukaryotic initiation factor eIF1 (83) and archaeal initiation factor aIF1 (84,85). Strikingly, IF3 and eIF1 both bind to both the 30S and 40S subunits in the same region (86). We suspect this conservation in binding will be of great interest in expanding dCasRx-IF3-mediated translational activation into organisms in diverse kingdoms of life.

Finally, we highlight the limitations of CRIPSR-RNAa in its current form. The strong differences between dCasRx and dCasRx-IF3 in activating gene expression required a carefully constructed setup, notably the removal of the inducer driving target mRNA transcription. In contexts more applicable to real-world use of such a tool, such as Supplementary Figure S3 or 5A where inducer was kept constant, fold-differences were 3.3 or 2.6 above basal levels and only 1.3 or 1.2 above enhancement caused by dCasRx-crRNA. Additionally, our data show that mRNAs with short 5′UTR may not be amenable to CRISPR-RNAa. The length of the 5′UTR in which RFP and LacZ were successfully activated were 59 and 53 nts respectively. The median 5′ UTR length of the 3746 mRNAs in *E. coli* is 153 nts, of which 29% are shorter than 53 nts and may not be amenable (87). Furthermore, given the apparent importance of targeting the start of the 5′UTR as shown in Figure 2, the ability to activate downstream genes with substantially longer 5′UTRs (such as those encoded downstream within an operon) remains to be seen. There is wide latitude for further optimizing CRISPR-RNAa, and future work will be required to address and overcome these limitations.

## DATA AVAILABILITY

The authors commit to providing the underlying data generated in this study upon reasonable request to the corresponding author.

## Supplementary Material

gkac680_Supplemental_File

## Notes

Present address: Peter Otoupal, Spotlight Therapeutics, Hayward, CA 94545, USA.
