# Supplementary material for: CRISPR-RNAa: targeted activation of translation using dCas13 fusions to translation initiation factors
Source: Nucleic Acids Res. 2022 Aug 11;50(15):8986–98. doi: 10.1093/nar/gkac680 (PMC9410913; doi:10.1093/nar/gkac680)
Supplement: gkac680_Supplemental_File [file gkac680_supplemental_file.pdf]

Supplementary Information to:

CRISPR-RNAa: Targeted Activation of Translation Using dCas13 Fusions to Translation Initiation Factors

Peter B. Otoupal, Brady F. Cress, Jennifer A. Doudna, and Joseph S. Schoeniger

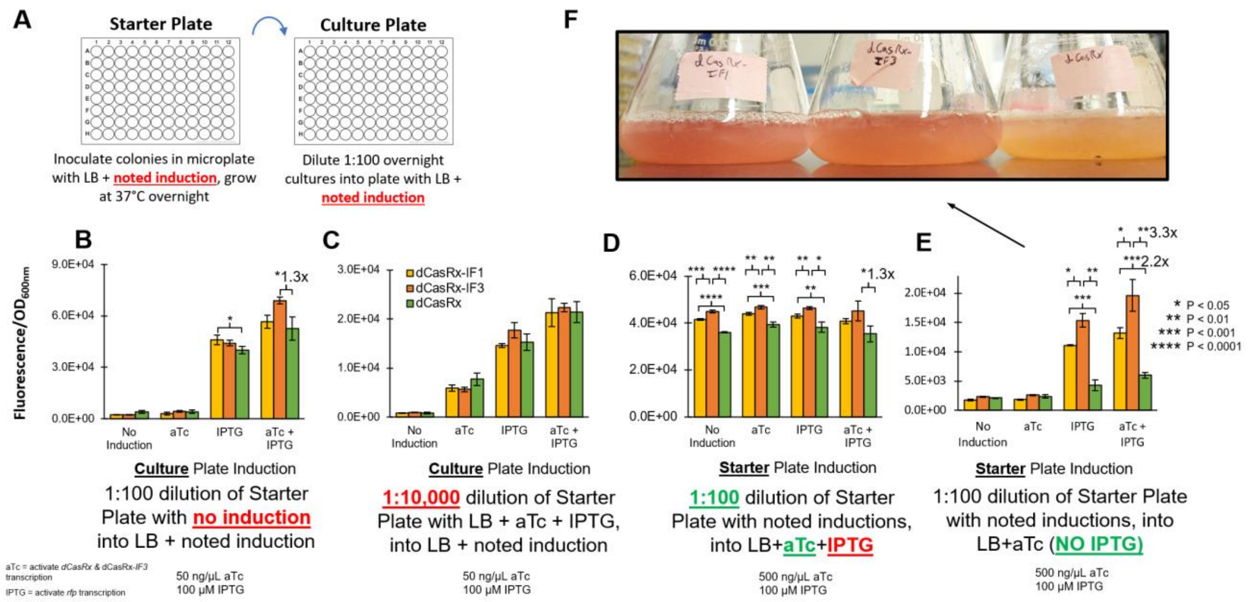

Figure S1. Process of optimizing experimental protocol for discerning influence of IF fusions to dCasRx. All error bars represent standard deviation of biological triplicates. (**A**) outline of experimental procedure. (**B**) Initial experimental procedure following that outlined in (**A**), showing slight enhancement of IF fusions. (**C**) Increasing dilution, along with pre-induction of the starter plate, resulted in no discernable fluorescence differences. (**D**) Keeping dilutions lower all resulted in fluorescence enhancements as in (**B**), regardless of the starter plate induction condition. (**E**) The optimal experimental setup was obtained when either IPTG was included in the initial starter plate, but removed in the culture plate. (**E**) and (**F**) represent the same data in Figure 1B and 1C. All error bars represent standard deviations of biological triplicates. Asterisks indicate *P*-values of two-tailed type II Student's *t*-tests.

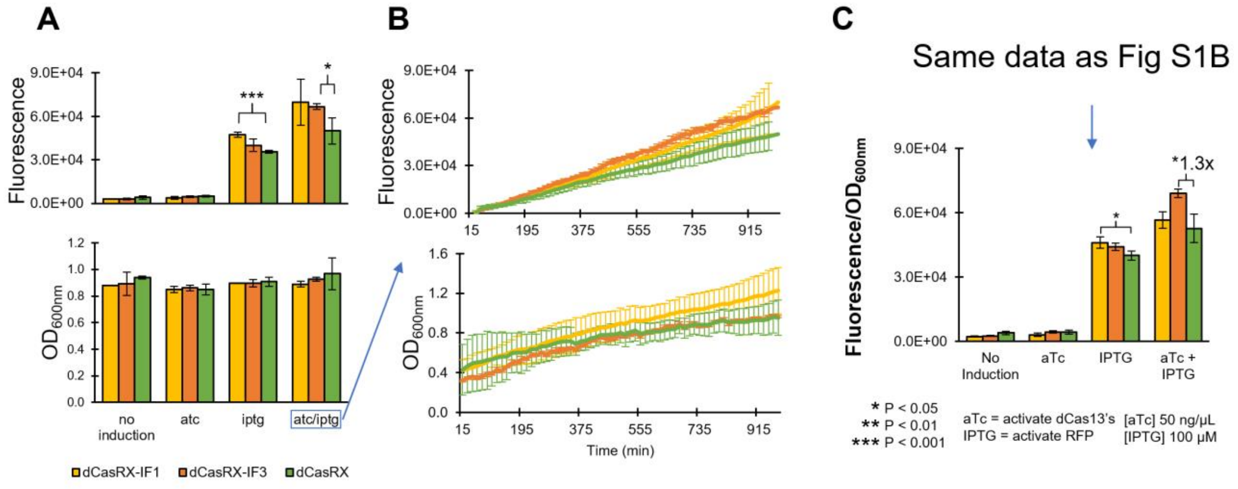

Figure S2. (**A**) Raw fluorescence and OD measurements of data from Figure S1B, as well as (**B**) kinetic growth and fluorescence profiles. (**C**) Same data as Figure S1B, wherein fluorescence is normalized to OD readings. All error bars represent standard deviations of biological triplicates. Asterisks indicate *P*-values of two-tailed type II Student's *t*-tests.

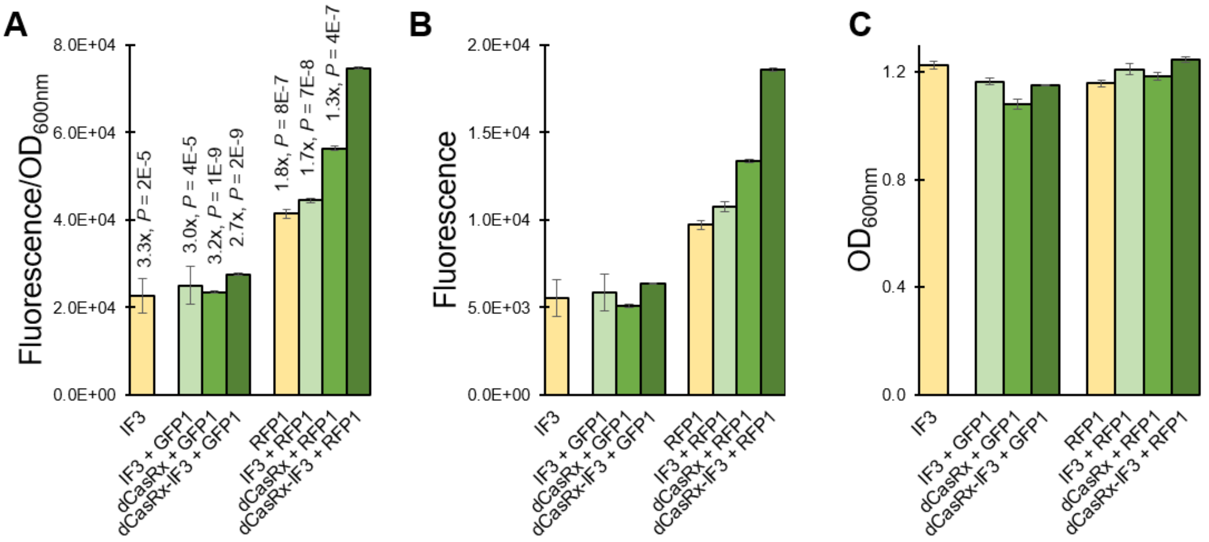

Figure S3. Effect of overexpressing Translation Initiation Factor IF3 alone, and with fusion to the C-terminal of dCasRx, alongside guide RNAs targeting a nonsense control (GFP-1) or the first 20 nt of the RFP 5'UTR (RFP-1). Individual colonies were inoculated into 500 µL LB without antibiotics, and grown overnight at 37°C. Samples were diluted 5 µL into 495 µL fresh LB containing 20 ng/µL aTc and 500 µM IPTG, grown at 37°C again overnight, and ending fluorescence and optical densities measured. (**A**) Fluorescence (arbitrary units) normalized to optical densities, as well as raw (**B**) fluorescence and (**C**) optical density values. In (**A**), fold differences of dCasRx-IF3 + RFP-1 relative to each other sample are shown above each respective bar, with associated *P*-value. A small yet statistically significant increase in expression was found between IF3 + RFP-targeting crRNA and the crRNA alone (1.07 ± 0.03, *P*=0.01), and was less than the increase when IF3 was replaced with dCasRx (1.36 ± 0.04, *P*=2E-5) or dCasRx-IF3 (1.80 ± 0.05, *P*=8E-7). Collectively, these data suggest the effect of IF3 overexpression itself was minimal. All error bars represent standard deviations of biological triplicates. *P*-values were calculated using a two-tailed type II Student's *t*-tests.

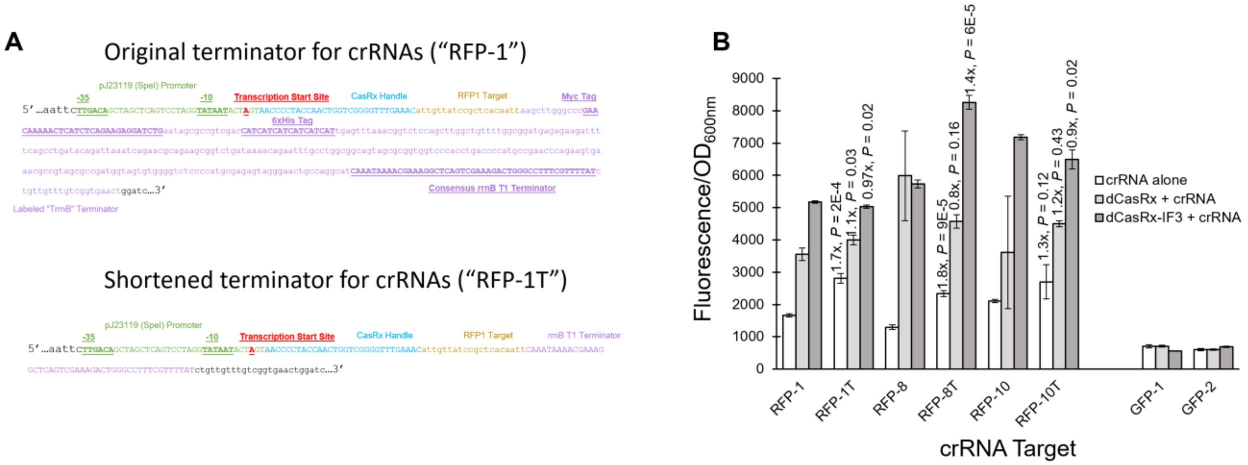

Figure S4. Effect of changing the terminator of the crRNA guide. (**A**) In our original design of the terminator, taken from a 367nt fragment labeled as “TrrnB terminator” from Table S2 of Chappell et al (Citation 44) (excluding the initial “G” nucleotide). While this terminator has worked well in previous studies, we noted that only the 46nt fragment near the end matches the consensus rrnB1 T1 terminator, while earlier sequences appear to code for the Myc and 6x-His tags. To ensure the crRNAs were not subject to influence from an extended 3’ end, we redesigned our crRNA terminator to shorten the 367nt TrrnB sequence to the consensus 46nt rrnB1 T1 sequence. All crRNAs using this redesigned terminator are annotated with a “T” at the end of their name to reflect this change. (**B**) Fluorescence of various length crRNAs targeting the start of RFP mRNA, either with the extended original terminator (RFP-1, RFP-8, RFP-10) or with the new shortened terminator (RFP-1T, RFP-8T, RFP-10T). Data was collected using the identical protocol used in Figure 2. Fold changes of the short terminator versus the original terminator are listed above each condition, with associated significance. All error bars represent standard deviations of biological triplicates. *P*-values were calculated using a two-tailed type II Student’s *t*-tests.

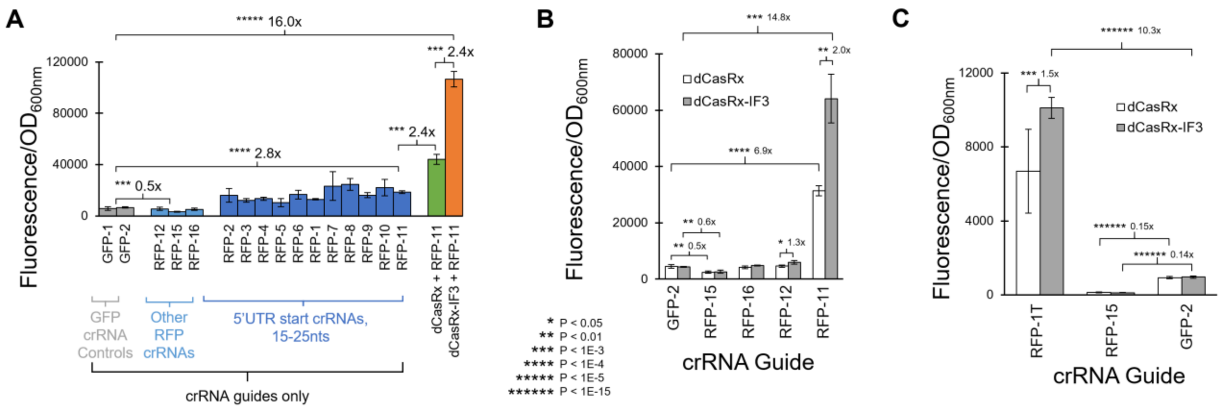

Figure S5. Three separate experiments repeating aspects of the data presented in Figure 2. (**A**) A repeat experiment focusing on a subset of crRNAs, and their comparison to dCasRx and dCasRx-IF3 with the RFP-11. Fluorescence comparison between strains harboring GFP-targeting crRNA guides (left, gray), and strains harboring various *rfp*-targeting crRNAs with and without dCasRx proteins. crRNAs targeting 10nt downstream of the 5'UTR start (RFP-12), the RBS (RFP-15), and 20 nt into the open reading frame of RFP (RFP-16) are shown in light blue (central left), and crRNAs targeting the beginning of the 5'UTR (RFP-1-11) with increasing length from 15 to 25 nt are shown in dark blue (central right). The 25 nt RFP-11 crRNA stacked with dCasRx or dCasRx-IF3 is shown on the right. (**B**) A repeat experiment utilizing dCasRx and dCasRx-IF3 with various crRNA targets. (**C**) A subset of strains from Figure 3 were repeated, using 3 biological replicates from 3 separate transformants (for a total of 9 biological replicates). All error bars represent standard deviations of biological triplicates (**A** and **B**) or nine biological replicates (**C**). Asterisks indicate P-values of two-tailed type II Student's *t*-tests.

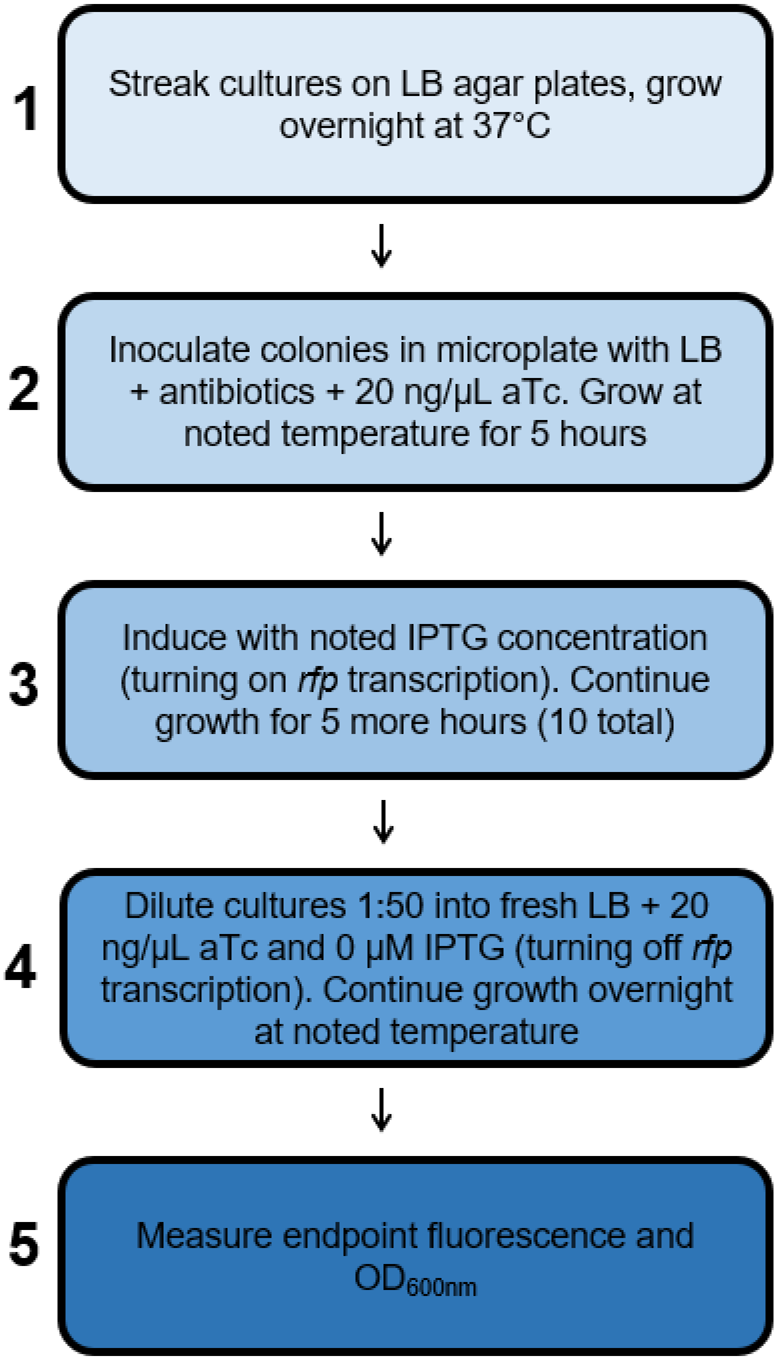

Figure S6. Revised experimental setup aimed at elucidating differences between dCasRx and dCasRx-IF3 strains. Relative to previous Figures 1 and 2, [aTc] (inducing dCas proteins' expression) was reduced from 500 ng/µL to 20 ng/µL, and IPTG induction was provided after 5 hours instead of at the start of the experiment. This new setup was used for Figures 3 and S7 at both 37°C and 30°C, and for Figures 4, 5, and S8-S13 at 30°C.

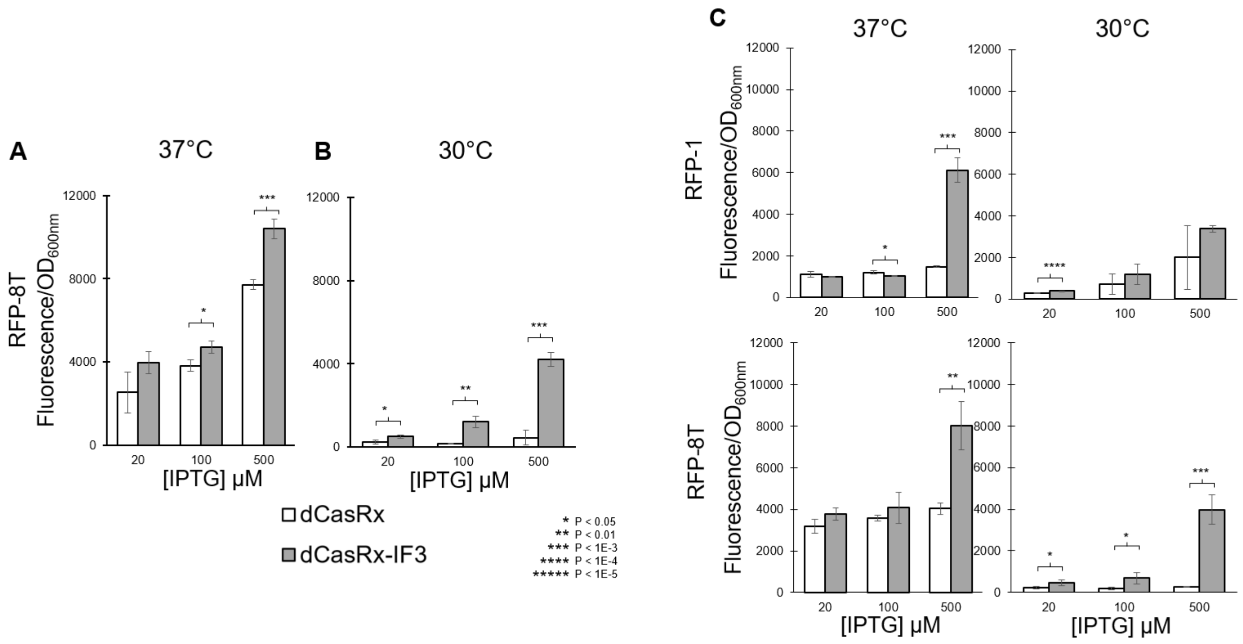

Figure S7. Variations in the experiment outlined in Figure 3. (**A**) and (**B**) are identical to Figure 3B and Figure 3C respectively, but utilize the 22nt RFP-8T crRNA in place of the 20nt RFP-1 crRNA. The "8T" designation indicates a redesign of the crRNA construct to use a shortened terminator as outlined in Figure S4. White bars represent dCasRx strains, while grey bars represent dCasRx-IF3 strains. (**C**) An experiment identical to that presented in Figure 3 and (**A**) and (**B**), but performed with IPTG induction at the start of the experiment instead of at 5 hours into the experiment (as outlined in Supplementary Figure S6). Fluorescence of strains harboring either protein alongside either the RFP-1 20nt crRNA (top) or the RFP-8T 22nt crRNA (bottom). The experiment was performed at either 37°C (left) or 30°C (right). All error bars represent standard deviation of biological triplicates. Asterisks indicate *P*-values of two-tailed type II Student's *t*-tests.

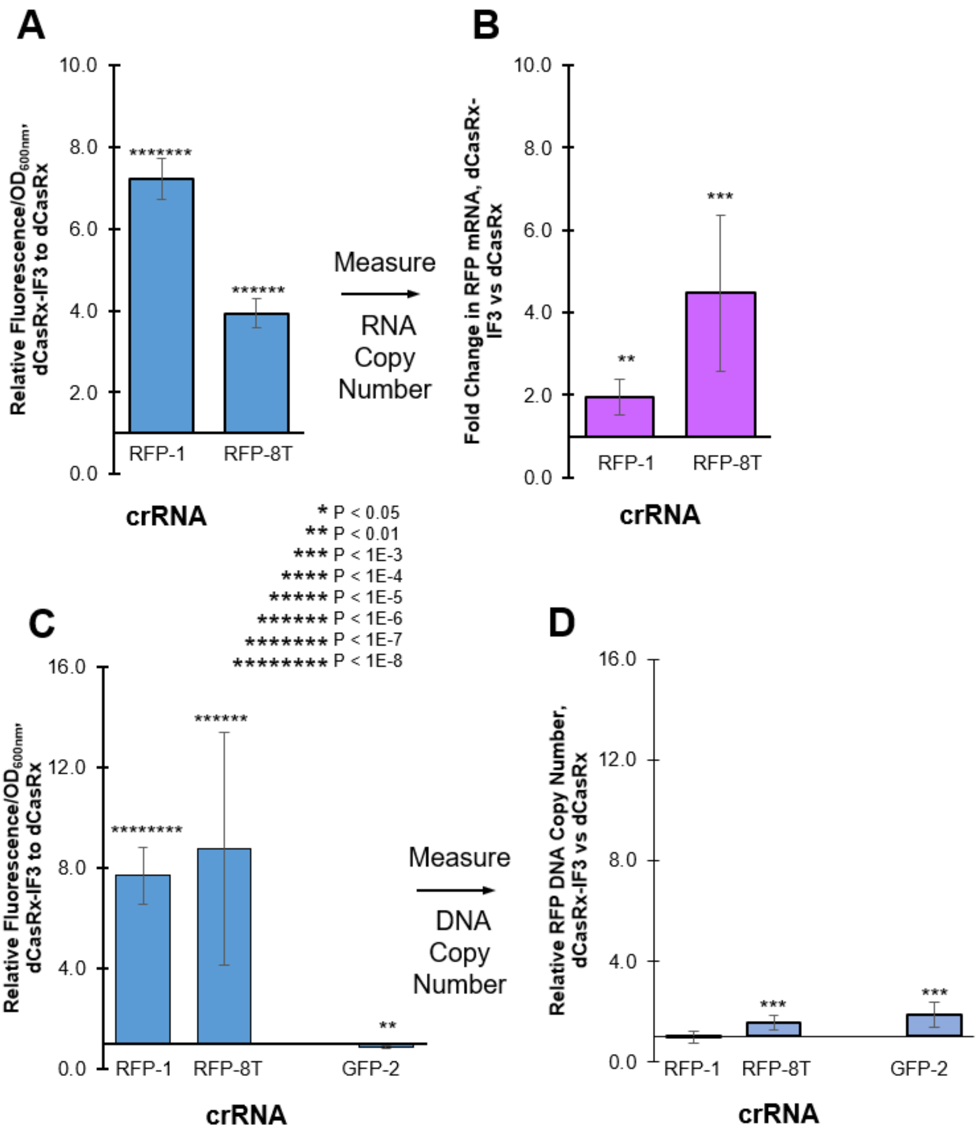

Figure S8. Measuring RNA and DNA copy number at time of fluorescence measurements, using the protocol outlined in Figure 3A. (**A**) Relative fluorescence of dCasRx-IF3 to dCasRx harboring two crRNA variants at the end of the experiment. (**B**) Relative copy number of *rfp* mRNA collected at the same time as fluorescence measurements in (**A**). (**C**) A repeat of the experiment A, to collect DNA copy number at the same time point as these fluorescence measurements. (**D**) Relative copy number of *rfp* DNA collected at the same time as fluorescence measurements in (**C**). All error bars represent standard deviation of four biological replicates. Asterisks indicate *P*-values of two-tailed type II Student's *t*-tests.

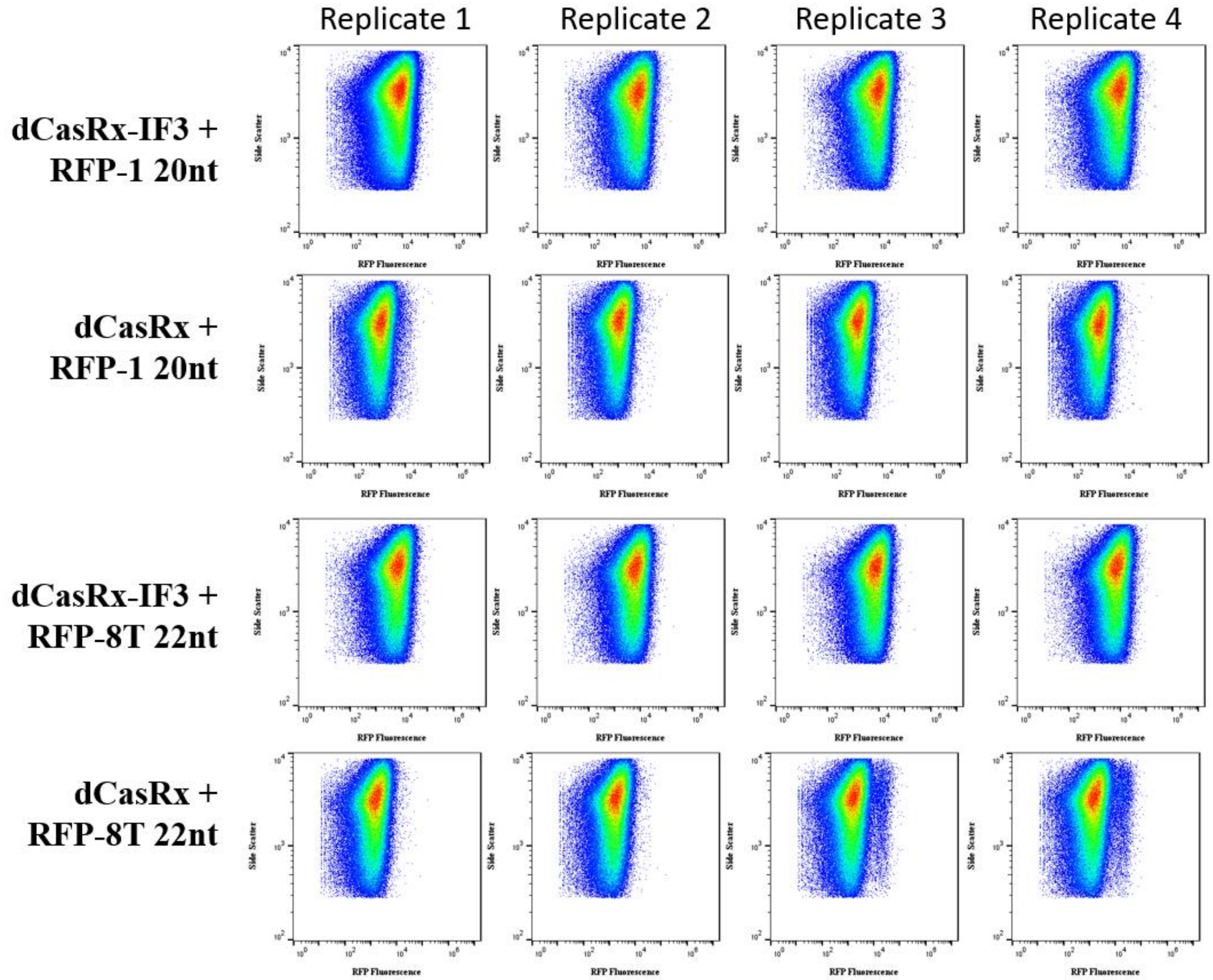

Figure S9. Flow cytometry of populations collected and analyzed at the same time point as data in Figure S8A and for qPCR analysis in Figure S8B. A minimum of approximately 500,000 events were collected, and gated using FlowJo® software. Data for each graph can be found in Supplementary Table S4.

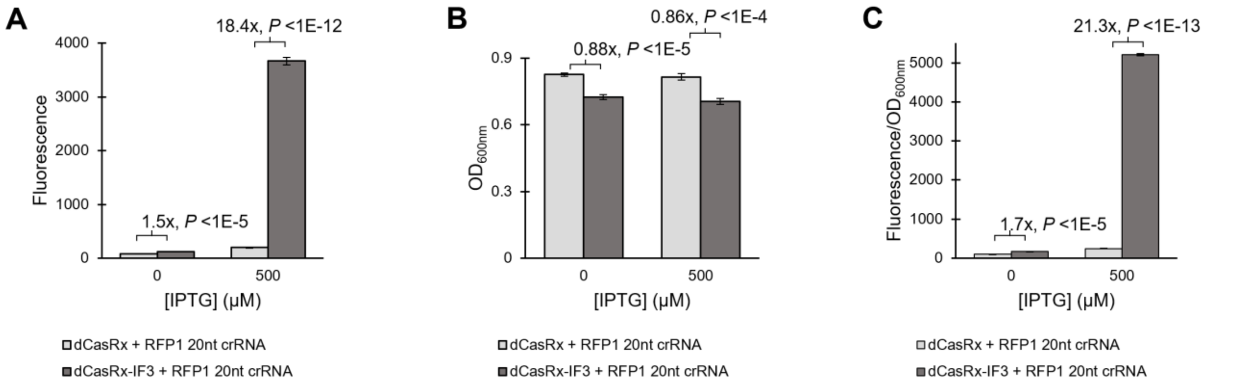

Figure S10. Raw (**A**) fluorescence, (**B**) OD_{600nm}, and (**C**) fluorescence normalized to OD_{600nm} measurements of data from Figure 4. All error bars represent standard deviations of four biological replicates. Statistical significance of *P*-values were calculated using two-tailed type II Student's *t*-tests.

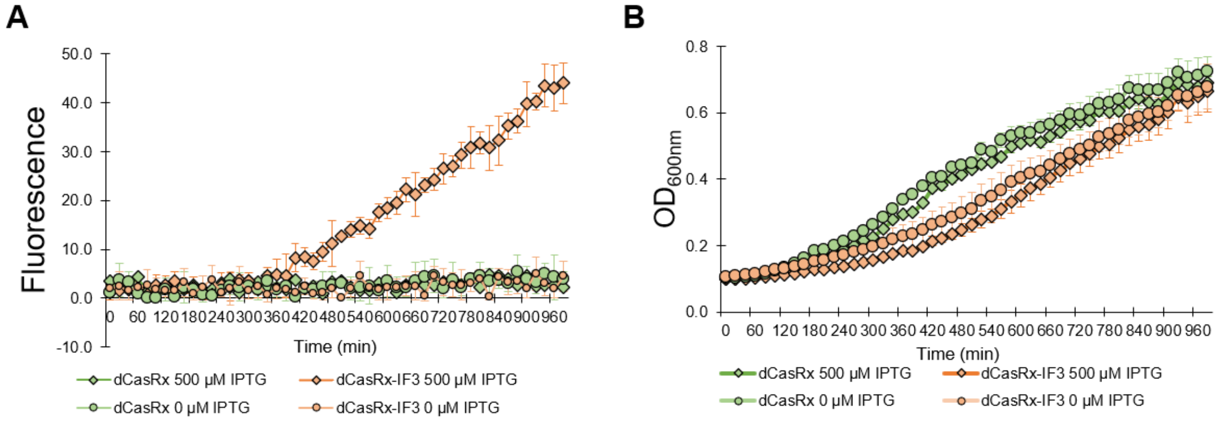

Figure S11. Online measurements performed in parallel with cultures used in Figure 4, where pre-cultures used to inoculate the 1 mL cultures used for Figure 4 for collecting RNA for RT-qPCR were also inoculated into the 100 µL cultures used here for online measurements (at step 4 as outlined in Supplementary Figure S6). (**A**) Fluorescence and (**B**) Optical density measurements overtime, measured every 20 minutes. Fluorescence measurements were performed using a gain of 50 instead of the usual gain of 100, resulting in lower overall measurements and higher background fluorescence. All error bars represent standard deviations of four biological replicates. Note that this experiment was performed online and was setup in parallel from pre-cultures used to inoculate cultures for Figure 4, and while reflective of the growth and fluorescence of cultures in Figure 4 over time, were cultivated with different shaking/aeration conditions.

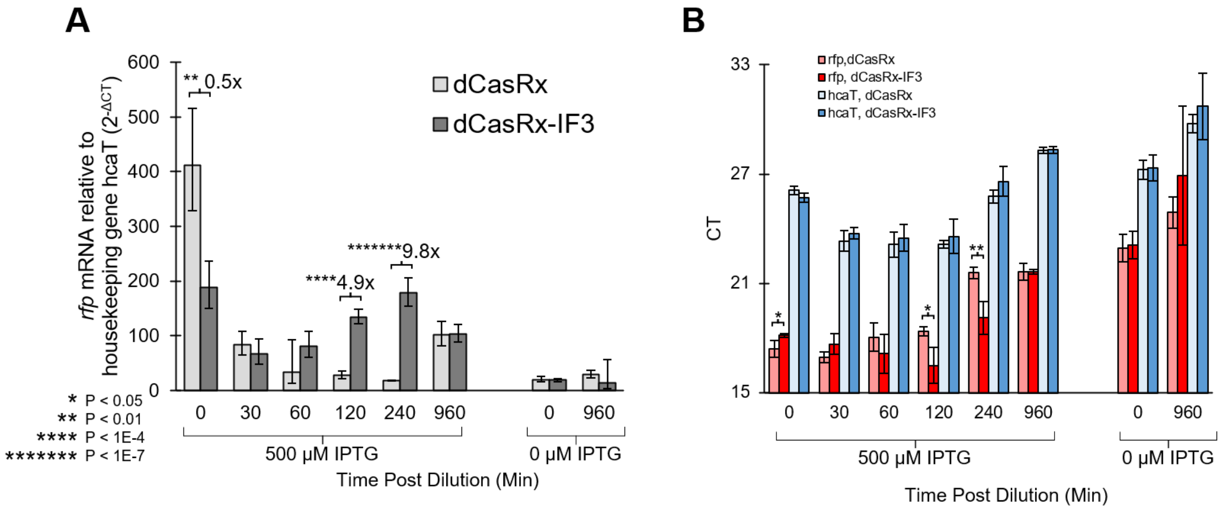

Figure S12. Raw data used for calculating relative *rfp* mRNA levels in Figure 4. (**A**) *rfp* mRNA relative to housekeeping gene *hcaT*, as calculated using the standard $2^{-\Delta CT}$ method. Time of collection post dilution into the final culture condition is noted, with IPTG induction in the pre-culture (left) and excluded (right). (**B**) CT values for all samples, based on four averages of biological replicates (themselves averages of two technical duplicates). Expression of *rfp* (red bars) and housekeeping *hcaT* (blue bars), in dCasRx strains (light red/blue) and dCasRx-IF3 strains (dark red/blue). The authors note that samples from time points 30, 60, and 120 min were prepared with twice as much starting cDNA (5 ng) than the remaining samples (2.5 ng), due to concerns of low overall RNA yield from extraction of low-density cultures. All error bars represent standard deviations of four biological replicates. Statistical significance of *P*-values were calculated using two-tailed type II Student's *t*-tests.

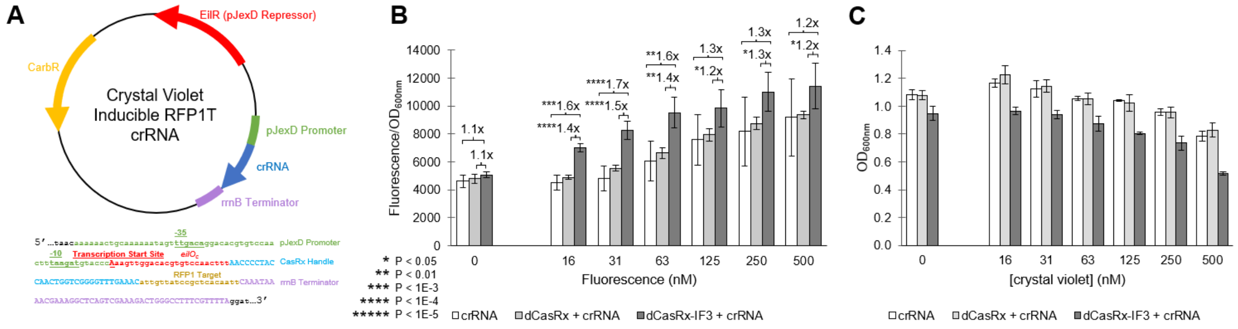

Figure S13. crRNAs can be made to be activated, and subsequently used to tune gene expression activation with dCasRx-IF3. (**A**) On our RFP-1T 20nt crRNA construct, we replaced the constitutively active pJ23119 (SpeI) promoter driving expression of our crRNA guide with the promoter pJexD, which has previously been shown to be inducible with crystal violet when coupled with the transcriptional repressor EilR (which binds to the operator *eilO*$_c$) with an EC_{50} of 120 nM. The sequence is listed below. We measured the ability of this system to activate expression of RFP with the crRNA alone, coupled with dCasRx, and coupled with dCasRx-IF3. (**B**) In the absence of crystal violet, no statistically significant differences in fluorescence were observed. With the lowest level crystal violet induction, only the strains harboring both the crRNA and dCasRx-IF3 increased in fluorescence. Increasing crystal violet concentration from here increased fluorescence in all strains, but (**C**) also led to a noticeable drop in growth as measured by optical densities. Background fluorescence was 26.5 ± 2.1 and 12.5 ± 3.5 in blank media containing 500 or 0 nM crystal violet respectively (technical duplicates), indicating negligible overlap of crystal violet with RFP fluorescence measurements. The experiment was performed at 30°C as outlined in Figure 3A. The significantly longer distance between the transcription start site and the CasRx handle versus our original promoter possibly contributed to lower overall activation differences from our previous experience with this setup. All error bars represent standard deviation of four biological replicates. Asterisks indicate *P*-values of two-tailed type II Student's *t*-tests.

Supplementary Table S1. Strains used in this study.

Strain Name	Base Strain	Description	Plasmid 1	Plasmid 2
P_ACT001	NEB 5-alpha	Just Expressing dCasRx	pPBO.ACT001	
P_ACT002	NEB 5-alpha	Just Expressing dCasRx-IF1	pPBO.ACT002	
P_ACT003	NEB 5-alpha	Just Expressing dCasRx-IF3	pPBO.ACT003	
P_ACT004	JBx_078907	RFP-1 15nt crRNA for targeting rfp 5'UTR start	pPBO.ACT007	
P_ACT005	JBx_078907	RFP-1 16nt crRNA for targeting rfp 5'UTR start	pPBO.ACT008	
P_ACT006	JBx_078907	RFP-1 17nt crRNA for targeting rfp 5'UTR start	pPBO.ACT009	
P_ACT007	JBx_078907	RFP-1 18nt crRNA for targeting rfp 5'UTR start	pPBO.ACT010	
P_ACT008	JBx_078907	RFP-1 19nt crRNA for targeting rfp 5'UTR start	pPBO.ACT011	
P_ACT009	JBx_078907	RFP-1 20nt crRNA for targeting rfp 5'UTR start	pPBO.ACT012	
P_ACT010	JBx_078907	RFP-1 21nt crRNA for targeting rfp 5'UTR start	pPBO.ACT013	
P_ACT011	JBx_078907	RFP-1 22nt crRNA for targeting rfp 5'UTR start	pPBO.ACT014	
P_ACT012	JBx_078907	RFP-1 23nt crRNA for targeting rfp 5'UTR start	pPBO.ACT015	
P_ACT013	JBx_078907	RFP-1 24nt crRNA for targeting rfp 5'UTR start	pPBO.ACT016	
P_ACT014	JBx_078907	RFP-1 25nt crRNA for targeting rfp 5'UTR start	pPBO.ACT017	
P_ACT015	JBx_078907	RFP-2 crRNA for targeting rfp 5'UTR, 10nt downstream of 5' UTR start	pPBO.ACT018	
P_ACT016	JBx_078907	RFP-RBS crRNA for targeting the RBS of rfp	pPBO.ACT019	
P_ACT017	JBx_078907	RFP-ORF crRNA for targeting inside the ORF of rfp	pPBO.ACT020	
P_ACT018	JBx_078907	GFP-1 crRNA for targeting gfp (control)	pPBO.ACT026	
P_ACT019	JBx_078907	GFP-2 crRNA for targeting gfp (control)	pPBO.ACT027	
P_ACT020	JBx_078907	RFP-1 15nt crRNA for targeting rfp 5'UTR start, with dCasRx	pPBO.ACT001	pPBO.ACT007
P_ACT021	JBx_078907	RFP-1 16nt crRNA for targeting rfp 5'UTR start, with dCasRx	pPBO.ACT001	pPBO.ACT008
P_ACT022	JBx_078907	RFP-1 17nt crRNA for targeting rfp 5'UTR start, with dCasRx	pPBO.ACT001	pPBO.ACT009
P_ACT023	JBx_078907	RFP-1 18nt crRNA for targeting rfp 5'UTR start, with dCasRx	pPBO.ACT001	pPBO.ACT010
P_ACT024	JBx_078907	RFP-1 19nt crRNA for targeting rfp 5'UTR start, with dCasRx	pPBO.ACT001	pPBO.ACT011
P_ACT025	JBx_078907	RFP-1 20nt crRNA for targeting rfp 5'UTR start, with dCasRx	pPBO.ACT001	pPBO.ACT012
P_ACT026	JBx_078907	RFP-1 21nt crRNA for targeting rfp 5'UTR start, with dCasRx	pPBO.ACT001	pPBO.ACT013
P_ACT027	JBx_078907	RFP-1 22nt crRNA for targeting rfp 5'UTR start, with dCasRx	pPBO.ACT001	pPBO.ACT014
P_ACT028	JBx_078907	RFP-1 23nt crRNA for targeting rfp 5'UTR start, with dCasRx	pPBO.ACT001	pPBO.ACT015
P_ACT029	JBx_078907	RFP-1 24nt crRNA for targeting rfp 5'UTR start, with dCasRx	pPBO.ACT001	pPBO.ACT016
P_ACT030	JBx_078907	RFP-1 25nt crRNA for targeting rfp 5'UTR start, with dCasRx	pPBO.ACT001	pPBO.ACT017
P_ACT031	JBx_078907	RFP-2 crRNA for targeting rfp 5'UTR, 10nt downstream of 5' UTR start, with dCasRx	pPBO.ACT001	pPBO.ACT018
P_ACT032	JBx_078907	RFP-RBS crRNA for targeting the RBS of rfp, with dCasRx	pPBO.ACT001	pPBO.ACT019
P_ACT033	JBx_078907	RFP-ORF crRNA for targeting inside the ORF of rfp, with dCasRx	pPBO.ACT001	pPBO.ACT020

Supplementary Table S1. Continued.

Strain Name	Base Strain	Description	Plasmid 1	Plasmid 2
P_ACT034	JBx_078907	RFP-1b 20nt crRNA for targeting RFP 5'UTR start, with a truncated crRNA 3' terminator region, with dCasRx	pPBO.ACT001	pPBO.ACT021
P_ACT035	JBx_078907	RFP-1b 22nt crRNA for targeting RFP 5'UTR start, with a truncated crRNA 3' terminator region, with dCasRx	pPBO.ACT001	pPBO.ACT022
P_ACT036	AG1	LacZ-1 crRNA for targeting lacZ 5'UTR, near Translation Start Site 3, with a truncated crRNA 3' terminator region, with dCasRx	pPBO.ACT001	pPBO.ACT023
P_ACT037	AG1	LacZ-2 crRNA for targeting lacZ 5'UTR, near Translation Start Site 2, with a truncated crRNA 3' terminator region, with dCasRx	pPBO.ACT001	pPBO.ACT024
P_ACT038	AG1	LacZ-3 crRNA for targeting lacZ 5'UTR, near Translation Start Site 4, with a truncated crRNA 3' terminator region, with dCasRx	pPBO.ACT001	pPBO.ACT025
P_ACT039	JBx_078907	GFP-1 crRNA for targeting gfp (control), with dCasRx	pPBO.ACT001	pPBO.ACT026
P_ACT040	JBx_078907	GFP-2 crRNA for targeting gfp (control), with dCasRx	pPBO.ACT001	pPBO.ACT027
P_ACT041	AG1	GFP-2 crRNA for targeting gfp (control), with dCasRx	pPBO.ACT001	pPBO.ACT027
P_ACT042	JBx_078907	RFP-1 20nt crRNA for targeting rfp 5'UTR start, with dCasRx-IF1	pPBO.ACT002	pPBO.ACT012
P_ACT043	JBx_078907	RFP-1 15nt crRNA for targeting rfp 5'UTR start, with dCasRx-IF3	pPBO.ACT003	pPBO.ACT007
P_ACT044	JBx_078907	RFP-1 16nt crRNA for targeting rfp 5'UTR start, with dCasRx-IF3	pPBO.ACT003	pPBO.ACT008
P_ACT045	JBx_078907	RFP-1 17nt crRNA for targeting rfp 5'UTR start, with dCasRx-IF3	pPBO.ACT003	pPBO.ACT009
P_ACT046	JBx_078907	RFP-1 18nt crRNA for targeting rfp 5'UTR start, with dCasRx-IF3	pPBO.ACT003	pPBO.ACT010
P_ACT047	JBx_078907	RFP-1 19nt crRNA for targeting rfp 5'UTR start, with dCasRx-IF3	pPBO.ACT003	pPBO.ACT011
P_ACT048	JBx_078907	RFP-1 20nt crRNA for targeting rfp 5'UTR start, with dCasRx-IF3	pPBO.ACT003	pPBO.ACT012
P_ACT049	JBx_078907	RFP-1 21nt crRNA for targeting rfp 5'UTR start, with dCasRx-IF3	pPBO.ACT003	pPBO.ACT013
P_ACT050	JBx_078907	RFP-1 22nt crRNA for targeting rfp 5'UTR start, with dCasRx-IF3	pPBO.ACT003	pPBO.ACT014
P_ACT051	JBx_078907	RFP-1 23nt crRNA for targeting rfp 5'UTR start, with dCasRx-IF3	pPBO.ACT003	pPBO.ACT015
P_ACT052	JBx_078907	RFP-1 24nt crRNA for targeting rfp 5'UTR start, with dCasRx-IF3	pPBO.ACT003	pPBO.ACT016
P_ACT053	JBx_078907	RFP-1 25nt crRNA for targeting rfp 5'UTR start, with dCasRx-IF3	pPBO.ACT003	pPBO.ACT017
P_ACT054	JBx_078907	RFP-2 crRNA for targeting rfp 5'UTR, 10nt downstream of 5' UTR start, with dCasRx-IF3	pPBO.ACT003	pPBO.ACT018
P_ACT055	JBx_078907	RFP-RBS crRNA for targeting the RBS of rfp, with dCasRx-IF3	pPBO.ACT003	pPBO.ACT019
P_ACT056	JBx_078907	RFP-ORF crRNA for targeting inside the ORF of rfp, with dCasRx-IF3	pPBO.ACT003	pPBO.ACT020

Supplementary Table S1. Continued.

Strain Name	Base Strain	Description	Plasmid 1	Plasmid 2
P_ACT057	JBx_078907	RFP-1b 20nt crRNA for targeting RFP 5'UTR start, with a truncated crRNA 3' terminator region, with dCasRx-IF3	pPBO.ACT003	pPBO.ACT021
P_ACT058	JBx_078907	RFP-1b 22nt crRNA for targeting RFP 5'UTR start, with a truncated crRNA 3' terminator region, with dCasRx-IF3	pPBO.ACT003	pPBO.ACT022
P_ACT059	AG1	LacZ-1 crRNA for targeting lacZ 5'UTR, near Translation Start Site 3, with a truncated crRNA 3' terminator region, with dCasRx-IF3	pPBO.ACT003	pPBO.ACT023
P_ACT060	AG1	LacZ-2 crRNA for targeting lacZ 5'UTR, near Translation Start Site 2, with a truncated crRNA 3' terminator region, with dCasRx-IF3	pPBO.ACT003	pPBO.ACT024
P_ACT061	AG1	LacZ-3 crRNA for targeting lacZ 5'UTR, near Translation Start Site 4, with a truncated crRNA 3' terminator region, with dCasRx-IF3	pPBO.ACT003	pPBO.ACT025
P_ACT062	JBx_078907	GFP-1 crRNA for targeting gfp (control), with dCasRx-IF3	pPBO.ACT003	pPBO.ACT026
P_ACT063	JBx_078907	GFP-2 crRNA for targeting gfp (control), with dCasRx-IF3	pPBO.ACT003	pPBO.ACT027
P_ACT064	AG1	GFP-2 crRNA for targeting gfp (control), with dCasRx-IF3	pPBO.ACT003	pPBO.ACT027

Supplementary Table S2. Plasmids used in this study.

Name	Description	Addgene ID
	**dCasRx plasmids**	
pPBO.ACT001	aTc inducible dCasRx	182711
pPBO.ACT002	aTc inducible dCasRx-IF1	182712
pPBO.ACT003	aTc inducible dCasRx-IF3	182713
pPBO.ACT004	Constitutively expressed crRNA cloning vector, extended 3' terminator region	182714
pPBO.ACT005	Constitutively expressed crRNA cloning vector, truncated 3' terminator region	182715
pPBO.ACT006	Constitutively expressed crRNA cloning vector, truncated 3' terminator region, with eGFP for cloning	182716
pPBO.ACT042	aTc inducible IF3 without dCasRx	N/A

Supplementary Table S2. Continued.

crRNA Plasmids

Name	Description	Spacer Sequence(5'-3')	Figure
pPBO.ACT007	RFP-2 15nt crRNA for targeting rfp 5'UTR start	uauccgcucacaauu	2,S5
pPBO.ACT008	RFP-3 16nt crRNA for targeting rfp 5'UTR start	uuauccgcucacaauu	2,S5
pPBO.ACT009	RFP-4 17nt crRNA for targeting rfp 5'UTR start	guuauccgcucacaauu	2,S5
pPBO.ACT010	RFP-5 18nt crRNA for targeting rfp 5'UTR start	uguuauccgcucacaauu	2,S5
pPBO.ACT011	RFP-6 19nt crRNA for targeting rfp 5'UTR start	uuguuauccgcucacaauu	2,S5
pPBO.ACT012	RFP-1 20nt crRNA for targeting rfp 5'UTR start	auuguuauccgcucacaauu	1,2,3,4,5,S1,S2,S3,S4,S5,S7,S8,S9,S10,S11,S12
pPBO.ACT013	RFP-7 21nt crRNA for targeting rfp 5'UTR start	aauuguuauccgcucacaauu	2,S5
pPBO.ACT014	RFP-8 22nt crRNA for targeting rfp 5'UTR start	aaauuguuauccgcucacaauu	2,S4,S5
pPBO.ACT015	RFP-9 23nt crRNA for targeting rfp 5'UTR start	gaaauuguuauccgcucacaauu	2,S5
pPBO.ACT016	RFP-10 24nt crRNA for targeting rfp 5'UTR start	ugaaauuguuauccgcucacaauu	2,S4,S5
pPBO.ACT017	RFP-11 25nt crRNA for targeting rfp 5'UTR start	cugaaauuguuauccgcucacaauu	2,S5
pPBO.ACT018	RFP-12 crRNA for targeting rfp, 10nt downstream the 5'UTR start	Gaauucugaaauuguuaucc	2,S5
pPBO.ACT019	RFP-13 crRNA for targeting rfp, 20nt downstream the 5'UTR start	aagaucuuuugaauucugaa	2
pPBO.ACT020	RFP-14 crRNA for targeting rfp, 30nt downstream the 5'UTR start	uccuucuuaaaagaucuuuu	2
pPBO.ACT021	RFP-15 crRNA for targeting rfp, 40nt downstream the 5'UTR start	uauguauaucuccuucuuaa	2,S5
pPBO.ACT022	RFP-16 crRNA for targeting rfp, 25nt downstream the RBS (near the start codon)	acgcaugaacucuuugauaa	2,S5
pPBO.ACT023	RFP-17 crRNA for targeting rfp, 335nt downstream the RBS (mRNA middle)	caccgucuugcagggaggag	2
pPBO.ACT024	RFP-18 crRNA for targeting rfp, 688nt downstream the RBS (mRNA 3' UTR)	agauccuuacucgaguuugg	2
pPBO.ACT025	RFP-1T 20nt crRNA for targeting rfp 5'UTR start, with short terminator	auuguuauccgcucacaauu	2,S4
pPBO.ACT026	RFP-8T 22nt crRNA for targeting rfp 5'UTR start, with short terminator	aaauuguuauccgcucacaauu	2,S4,S8,S9
pPBO.ACT027	RFP-10T 24nt crRNA for targeting rfp 5'UTR start, with short terminator	aaauuguuauccgcucacaauu	2,S4
pPBO.ACT028	RFP-19T 20nt crRNA for targeting 1nt from rfp 5'UTR start, with short terminator	auugugagcggauaacaauu	2
pPBO.ACT029	RFP-20T 20nt crRNA for targeting 2nt from rfp 5'UTR start, with short terminator	uugugagcggauaacaauuu	2
pPBO.ACT030	RFP-21T 20nt crRNA for targeting 3nt from rfp 5'UTR start, with short terminator	ugugagcggauaacaauuuc	2
pPBO.ACT031	RFP-22T 20nt crRNA for targeting 4nt from rfp 5'UTR start, with short terminator	gugagcggauaacaauuuca	2
pPBO.ACT032	RFP-23T 20nt crRNA for targeting 5nt from rfp 5'UTR start, with short terminator	ugagcggauaacaauuucag	2
pPBO.ACT033	RFP-24T 20nt crRNA for targeting 6nt from rfp 5'UTR start, with short terminator	gagcggauaacaauuucaga	2
pPBO.ACT034	RFP-25T 20nt crRNA for targeting 7nt from rfp 5'UTR start, with short terminator	agcggauaacaauuucagaa	2
pPBO.ACT035	RFP-26T 20nt crRNA for targeting 8nt from rfp 5'UTR start, with short terminator	gcggauaacaauuucagaau	2
pPBO.ACT036	RFP-27T 20nt crRNA for targeting 9nt from rfp 5'UTR start, with short terminator	cggauaacaauuucagaauu	2
pPBO.ACT037	LacZ-1T crRNA for targeting lacZ 5'UTR, near Translation Start Site 3, with short terminator	gcucacaauuccacacaacauacga	5
pPBO.ACT038	LacZ-2T crRNA for targeting lacZ 5'UTR, near Translation Start Site 2, with short terminator	auuccacacaacauacgagccggaa	5
pPBO.ACT039	LacZ-3T crRNA for targeting lacZ 5'UTR, near Translation Start Site 4, with short terminator	auacgagccggaagcauaaagugua	5
pPBO.ACT040	GFP-1 crRNA for targeting gfp (control)	ugggacaacuccagugaaaa	2,S3,S4,S5
pPBO.ACT041	GFP-2 crRNA for targeting gfp (control)	gauacgauuaacaaggguau	2,3,5,S4,S5,S8
pPBO.ACT043	RFP-1 20nt crRNA for targeting rfp 5'UTR start, under pJexD crystal violet promoter	auuguuauccgcucacaauu	S13

Supplementary Table S3. Primers used in this study.

Plasmid Construction Primers

Name	Sequence	Purpose
PBO.ACT001	gtccggcaattccggtggaggaggctctg	Amplifying IF1 and IF3 gBlocks, Fwd
PBO.ACT002	tatttgatgccctattattacttgaagcgatatgtgatgcg	Amplifying IF1 gBlock, Rev
PBO.ACT003	cgttttatttgatgccctattattactgcttttttttgggagcc	Amplifying IF3 gBlock, Rev
PBO.ACT004	gcatcacatatcgcttcaagtaataatagggcatcaaataaaacgaaagg	Amplifying dCasRx for Gibson Assembly
PBO.ACT005	tcatgtaagatcctttctcctctttagatcttttgaattcttttctc	Amplifying dCasRx for Gibson Assembly
PBO.ACT006	ctaaagaggagaaaggatcttacatgatcgaaaaaaaaaagtcc	Amplifying dCasRx for Gibson Assembly
PBO.ACT007	gagcctcctccaccggaattgccggacacc	Amplifying dCasRx for Gibson Assembly
PBO.ACT008	ggctcccaaaaaaaagcagtaataatagggcatcaaataaaacgaaagg	Amplifying dCasRx for Gibson Assembly
PBO.ACT009	gaatatggctcattttagcttccttagctcctgaaaatctc	Amplifying dCasRx for Gibson Assembly
PBO.ACT010	gctaaggaagctaaaatgagccatattcaacggg	Amplifying dCasRx for Gibson Assembly
PBO.ACT011	cgatatcaaattagaaaaactcatcgagcatc	Amplifying dCasRx for Gibson Assembly
PBO.ACT012	cgatgagtttttctaatttgatatcgagctcgcttgg	Amplifying dCasRx for Gibson Assembly
PBO.ACT013	cggagacgaacgtctcaaagcttgggcccgaac	Amplifying crRNA backbone plasmid, for pPBO.ACT004
PBO.ACT014	cccaagctttgagacgttcgtctccgtttcaaaccc	Amplifying crRNA backbone plasmid, for pPBO.ACT004
PBO.ACT015	gtcttaccgggttggactcaagacgatagt	Amplifying crRNA backbone plasmid, for pPBO.ACT004-6
PBO.ACT016	actatcgtcttgagtccaacccggtaagac	Amplifying crRNA backbone plasmid, for pPBO.ACT004-6
PBO.ACT017	ggagacgaacgtctcacaaataaaacgaaaggctca	Amplifying crRNA backbone plasmid, for pPBO.ACT005
PBO.ACT018	agcctttcgttttatttgtgagacgttcgtctcc	Amplifying crRNA backbone plasmid, for pPBO.ACT005
PBO.ACT019	aaacggagacgaccggcattggtcttcagac	Amplifying crRNA backbone plasmid, for pPBO.ACT006
PBO.ACT020	gaactgtacaaatgaacgtctcacaaataaaacgaaaggct	Amplifying crRNA backbone plasmid, for pPBO.ACT006
PBO.ACT021	ttatttgtgagacgttcatttgtacagttcatccatacc	Amplifying crRNA backbone plasmid, for pPBO.ACT006
PBO.ACT022	gaagaccaatgccggtcgtctccgtttcaaaccccg	Amplifying crRNA backbone plasmid, for pPBO.ACT006
PBO.ACT023	gaagaccaatgccggtcgtctccgtttcaaaccccg	Removing dCasRx from pPBO.ACT003 to make pPBO.ACT042
PBO.ACT024	gaagaccaatgccggtcgtctccgtttcaaaccccg	Removing dCasRx from pPBO.ACT003 to make pPBO.ACT042
PBO.ACT025	gaaaggatcttacatgaaaggaggaaaacgcgttc	Creating pPBO.ACT043 from pPBO.ACT012
PBO.ACT026	cgttttcctcctttcatgtaagatcctttctcctctttagatc	Creating pPBO.ACT043 from pPBO.ACT012
PBO.ACT027	tgtacccaaagttggacacgtgtccaactttaacccctaccaactggtcg	Creating pPBO.ACT043 from pPBO.ACT012
PBO.ACT028	tgtcaaactatttttttgcagtttttttgaattccagaaatcatccttagcg	Creating pPBO.ACT043 from pPBO.ACT012
PBO.ACT029	gacaggacacgtgtccaactttaagatgtacccaaagttggacacgtgtccaactttaac	Creating pPBO.ACT043 from pPBO.ACT012
PBO.ACT030	atcttaaagttggacacgtgtcctgtcaaactatttttttgcagttttttg	Creating pPBO.ACT043 from pPBO.ACT012

Supplementary Table S3. Continued

Sequencing Primers

Name	Sequence	Purpose
PBO.SEQ001	taagcagctctaatgcgc	Sequencing dCasRx constructs
PBO.SEQ002	ggctctacaacctcgataag	Sequencing dCasRx constructs
PBO.SEQ003	tatgatgagctcaaggccc	Sequencing dCasRx constructs
PBO.SEQ004	cgcctatatcaacgacattgc	Sequencing dCasRx constructs
PBO.SEQ005	gttcctgcctttcggtttacc	Sequencing dCasRx constructs
PBO.SEQ006	acttgttcaacaggccagc	Sequencing dCasRx constructs
PBO.SEQ007	gatttcgtgatgcttgtcaggg	Sequencing dCasRx constructs
PBO.SEQ008	gcgacacggaaatgttgaatact	Sequencing crRNA constructs
PBO.SEQ009	tttgagtgagctgataccgctcg	Sequencing crRNA constructs

qPCR Primers

Name	Sequence	Purpose
PBO.qPCR001	ctgccgggtgcttacaaaac	qPCR of RFP, Fwd. Amplicon length 90bp, 500nM working concentration
PBO.qPCR002	ttaagcaccggtggagtgac	qPCR of RFP, Rev. Amplicon length 90bp, 500nM working concentration
PBO.qPCR003	gacgggcaaactggtcacta	qPCR of hcat housekeeping gene, Fwd. Amplicon length 114bp, 500nM working concentration
PBO.qPCR004	atgagaaagccgagcagcat	qPCR of hcat housekeeping gene, Rev. Amplicon length 114bp, 500nM working concentration

IDT gBlocks

Name	Sequence	Purpose
3xGGGS - *infA*	ggtggaggaggctctggtggaggcggtagcggaggcggagggtcggccaaagaa<br>gatgtcatcgaaattgaaggcaaagttgtagaaaccatgcccaacgctatgttt<br>acagtggaacttgagaacgggcatcagattttagcgaccgtaagcgggaaaatc<br>cgcaaaaactatatccgtatccttgtcggtgacaaggtgacggttgaaatgagc<br>ccctatgatttgacgcgtgggcgcatcacatatcgcttcaagtaa	gBlock to attach IF1 onto dCasRx C-term
3xGGGS - *infC*	ggtggaggaggctctggtggaggcggtagcggaggcggagggtcgaaaggagga<br>aaacgcgttcagaccgcacgccctaaccgcattaacggcgagattcgcgcacag<br>gaggttcgcctgacggggttagagggtgagcaacttggtatcgtgagcttgcgc<br>gaggcgttggaaaaagctgaagaagccggtgtcgacttagtagagattagtcct<br>aacgccgagccacccgtgtgccgcatcatggactatggtaaattcctgtatgaa<br>aagagtaaatcaagcaaagaacagaaaaagaagcaaaaggtgattcaagtaaaa<br>gagattaaatttcgcccgggtacggatgagggcgactaccaggtcaaattgcgt<br>tcacttattcgttttcttgaggaaggagacaaggctaaaattacgttacgcttt<br>cgcggtcgcgagatggcacaccaacaaatcgggatggaagtcttgaaccgcgta<br>aaagatgatttacaagagttagctgtagtcgagagttttcctacaaaaattgaa<br>ggtcgccaaatgatcatggttttggctcccaaaaaaaagcag	gBlock to attach IF3 onto dCasRx C-term

Supplementary Table S4. FACS data from Supplementary Figure S9.

	Rep:	Count	Median Forward Scatter	CV Forward Scatter	Median Side Scatter	CV Side Scatter	Median RFP Fluorescence	CV RFP Fluorescence
**dCasRx-IF3 + RFP-1 20nt**	1	300408	4041	46.1	2313	64.8	5312	94.4
	2	157416	4036	45.7	2209	65.6	5333	92.7
	3	158779	4054	46.1	2382	64.0	6025	92.9
	4	156813	4091	46.1	2382	64.7	5668	93.1
**dCasRx + RFP-1 20nt**	1	133360	4250	47.7	2398	62.1	897	119.0
	2	135371	4250	48.1	2548	61.0	868	91.8
	3	134712	4207	48.0	2475	61.9	868	91.2
	4	133565	4250	47.6	2342	62.5	872	94.0
**dCasRx-IF3 + RFP-1b 22nt**	1	158112	4041	45.8	2212	65.6	5290	86.7
	2	158027	4041	45.7	2119	66.2	4917	86.6
	3	158237	4050	45.4	2112	65.8	5600	85.4
	4	157902	4027	45.9	2182	65.6	5510	85.5
**dCasRx + RFP-1b 22nt**	1	144873	4212	47.0	2417	63.4	1256	94.8
	2	148458	4151	47.4	2401	63.3	1425	94.6
	3	148732	4203	47.0	2508	62.4	1368	179.0
	4	144585	4269	47.6	2537	61.9	1272	188.0
